# Current Progress and Challenges of Electromagnetic Wave Absorbing Materials at High Temperature

**DOI:** 10.1002/advs.202504286

**Published:** 2025-06-05

**Authors:** Zhixiao Wang, Tao Yang, Linlin Zhou, Xinmei Hou, Zhi Fang, Yanglong Hou

**Affiliations:** ^1^ Institute for Carbon Neutrality University of Science and Technology Beijing Beijing 100083 China; ^2^ Liaoning Academy of Materials Shenyang 110000 China; ^3^ School of Materials Shenzhen Campus of Sun Yat‐sen University Shenzhen 518107 China; ^4^ State Key Laboratory of Optoelectronic Materials and Technologies Sun Yat‐sen University Guangzhou 510275 China; ^5^ School of Materials Science and Engineering Peking University Beijing 100871 China

**Keywords:** electromagnetic loss mechanism, electromagnetic wave absorption;^,^high temperature, SiC, testing method

## Abstract

With the rapid development of science and technology, electromagnetic waves have deeply penetrated various fields. However, they also bring a higher demand for electromagnetic protection. Electromagnetic wave absorbing materials (EMWAMs) can significantly reduce or even eliminate the reflection and scattering of electromagnetic waves by converting electromagnetic wave energy into heat energy and dissipation. In recent years, the research work on EMWAMs mainly focuses on the material systems applied at the normal temperature, and its absorption mechanism is systematically discussed. However, at the elevated temperature, the electromagnetic loss mechanism changes, and the suitable EMWAMs become limited. At the same time, the testing method of EMWAMs at high temperature also remains a challenge. In this work, the electromagnetic loss mechanism and testing methods for high‐temperature EMWAMs, especially above 800 °C, are discussed. Based on this, a selection criterion for EMWAMs at high temperature is proposed.

## Graphical abstract

This review explains three dielectric loss mechanisms of electromagnetic wave absorbing materials under high temperatures and the high temperature testing technology is explained. The research progress of carbon‐based, ceramic‐based and metamaterial‐based electromagnetic wave absorbers is analyzed. It is finally concluded that SiC ceramics and interfacial polarization loss mechanism are the key directions in this field in the future.

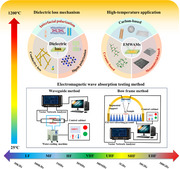

## 1 Introduction

The pervasive application of electromagnetic waves is widespread in modern society.^[^ ^1^ ^]^ For example, low‐frequency (LF) electromagnetic waves (30–300 kHz) are primarily used for communication in the atmosphere or ocean, capitalizing on their stronger penetration ability.^[^ ^2^ ^]^ The medium‐frequency (MF, from 300 kHz to 3 MHz), high‐frequency (HF, 3–30 MHz), very high frequency (VHF, 30–300 MHz), and ultrahigh frequency (UHF, from 300 MHz to 3 GHz) electromagnetic waves are mainly utilized for heating and communication.^[^ ^3^ ^]^ In the super‐high‐frequency (SHF, 3–30 GHz) range, electromagnetic wave offers high resolution, making them ideal for military radar and medical imaging. As the frequencies reach extremely high (EHF, 30–300 GHz), they are mainly used for satellite communications and material testing.^[^ ^4^, ^5^ ^]^ Concurrently, the electromagnetic hazards caused by these waves have garnered increasing attention.^[^ ^6^, ^7^ ^]^ The existence of electromagnetic waves will reduce the survival rate of military equipment and pose a potential risk to the environment and human health. As an effective solution, electromagnetic wave absorbing materials (EMWAMs) can convert electromagnetic wave energy into other forms of energy. In the military field, EMWAMs, as an indispensable part of stealth technology, can effectively attenuate electromagnetic wave energy, decrease the reflection of radar signals, significantly decrease the detectability of military equipment such as fighter jets, naval vessels, and missiles, and improve their battlefield survivability. In the civil field, EMWAMs can effectively mitigate the impact of electromagnetic radiation on human health and minimize interference among electronic devices.^[^ ^8^, ^9^, ^10^, ^11^, ^12^, ^13^ ^]^

In recent years, EMWAMs have been vigorously developed for various scenarios, and their electromagnetic wave absorption mechanisms have been deeply explored.^[^ ^14^, ^15^, ^16^, ^17^, ^18^, ^19^ ^]^ However, with the development of military equipment and civil technology, stricter requirements are put forward for EMWAMs, especially in high‐temperature service environments.^[^ ^20^, ^21^ ^]^ Compared with the research of EMWAMs at normal temperature, progress in high‐temperature EMWAMs especially above 800 °C remains in its nascent stages.^[^ ^22^ ^]^ However, electromagnetic protection of aerospace hot‐end components (e.g., engine nozzles and turbine blades) urgently needs EMWAMs that can be used at temperatures above 800 °C. The main reasons can be attributed to the following three aspects: first, the electromagnetic loss mechanism at high temperature lacks a comprehensive analysis. Second, the testing methods and in situ characterization technologies for assessing the electromagnetic wave absorbing performance in high‐temperature environments are still immature. Finally, magnetic materials are not suitably applied above 800 °C due to the limitation of the Curie temperature. Suitable materials requiring both the chemical stability and physical properties of materials under high‐temperature conditions are greatly restricted.

In this work, we systematically summarize the electromagnetic wave loss mechanism especially under high‐temperature conditions, focusing on the dielectric loss mechanisms of materials. Subsequently, we discuss the testing technologies for high‐temperature electromagnetic wave absorbing performance, especially the related in situ characterization methods. Finally, the design and selection criteria of high‐temperature absorbing material systems are discussed, including the performance of materials at high temperatures and their optimization strategies. Combining current research progress, the future development of high‐temperature EMWAMs is put forward.

## 2 The Electromagnetic Loss Mechanism of EMWAMs at High Temperature

When electromagnetic waves reach the surface of the EMWAMs, part of the waves will enter the material, while the other part will be reflected into the atmosphere.^[^ ^23^ ^]^ The electromagnetic waves that enter the material will interact with the material thereby converting part of the electromagnetic energy into heat energy. Simultaneously, the waves inside the material will undergo multiple scattering and reflections. Eventually, the electromagnetic waves that cannot be absorbed will pass through the EMWAM and are transmitted. Therefore, material with excellent electromagnetic wave absorbing performance must fulfill two key requirements, i.e., excellent impedance matching character and high electromagnetic wave attenuation ability, which can quickly convert the electromagnetic energy inside the absorbing material into heat energy or other forms of energy.^[^ ^24^ ^]^ According to the electromagnetic wave transmission line theory, the impedance of the absorbing material *Z*_in_ can be expressed by Formula (1).^[^ ^25^ ^]^ In general, the reflection loss (RL) is commonly used to evaluate the electromagnetic wave absorption characteristics of EMWAMs, expressed by the following formula (Equation (2))

(1) $$Z_{\mathrm{in}}=Z_{o}{\left(\mu_{r}/\varepsilon_{r}\right)}^{1/2}\tan\;\left[j\left(2\pi fd/c\right){\left(\mu_{r}\varepsilon_{r}\right)}^{1/2}\right]$$

(2) $$\mathrm{RL}\left(\mathrm{dB}\right)=20\;log_{10}\left|\frac{Z_{\mathrm{in}}-Z_{0}}{Z_{\mathrm{in}}+Z_{0}}\right|$$

where *Z*_in_ is the normalized impedance of the material, *Z*_0_ is the air impedance, *𝜀*_𝑟_ is the material's complex dielectric constant, *𝜇*_𝑟_ is the complex permeability of the material, 𝑓 is the frequency of the electromagnetic wave, 𝑑 is the thickness of the material, 𝑐 is the speed of light, and 𝑗 is the imaginary unit. When *Z*_in_ = *Z*_0_, namely the normalized impedance of the material and the impedance of the air are equal, electromagnetic waves can completely enter the material. Attenuation loss is used to describe the energy consumption of electromagnetic energy converted into heat or other forms as it enters the material. The attenuation loss coefficient can be expressed by the following formula (Equation (3))^[^ ^26^ ^]^

(3) $$\alpha =\frac{\sqrt{2\pi f}}{c}\times \sqrt{\left(\mu^{\prime \prime }\varepsilon^{\prime \prime }-\mu^{\prime }\varepsilon^{\prime }\right)+\sqrt{{\left(\mu^{\prime \prime }\varepsilon^{\prime \prime }-\mu^{\prime }\varepsilon^{\prime }\right)}^{2}+{\left(\mu^{\prime \prime }\varepsilon^{\prime \prime }+\mu^{\prime }\varepsilon^{\prime }\right)}^{2}}}$$

where *α* is the attenuation coefficient, *𝜀*′ is the real part of the complex dielectric constant, *𝜀*″ is the imaginary part of the complex dielectric constant, *𝜇*′ is the real part of the complex permeability, and *𝜇*″ is the imaginary part of the complex permeability. Other symbols retain their previous meanings as defined above.

From Formulas (1)–(3), it can be seen that when *𝜇*″ and *𝜀*″ increase, *𝜇*′ and *𝜀*′ decrease, and the attenuation coefficient *α* becomes larger, enhancing the material's ability to dissipate electromagnetic waves. To attain the optimal electromagnetic wave absorption characteristics, it is necessary to simultaneously obtain excellent impedance matching and the highest possible attenuation loss. However, achieving high losses and optimal impedance matching for a given material are mutually exclusive and cannot be optimized simultaneously. Consequently, the coordination of attenuation loss and impedance matching represents a critical aspect of achieving effective electromagnetic wave absorption.^[^ ^27^ ^]^

The core challenge in the design of high‐temperature EMWAMs is still to balance the two contradictory goals of attenuation loss and impedance matching. In high‐temperature environments, the range of material selection is significantly narrowed due to thermal stability limitations, and the magnetic loss mechanism of magnetic materials fails due to Curie temperature limitations, which further aggravates the difficulty of collaborative optimization between the two. The selection of material systems and the synergistic construction of a high‐temperature stable loss mechanism are very effective means.

### 2.1 Magnetic Loss Mechanism

The magnetic loss mechanism plays an important role in EMWAMs, which can effectively control impedance matching.^[^ ^28^, ^29^, ^30^, ^31^ ^]^ It is mainly related to the absorption of incident electromagnetic wave energy through the hysteresis effect of materials via magnetic field, magnetic polarization, and magnetic resonance mechanism, respectively, corresponding to hysteresis loss, eddy current loss, and domain wall resonance.^[^ ^32^, ^33^, ^34^, ^35^ ^]^

The Curie temperature marks the transition point at which a substance shifts from exhibiting ferromagnetic behavior to a paramagnetic state, below which it retains its ferromagnetic properties. Typically, to avoid high‐temperature demagnetization and performance degradation, magnetic materials are operated within a temperature range of 50–70% of their Curie temperature.

The maximum Curie temperature of magnetic materials is 850 °C.^[^ ^36^ ^]^ However, in order to ensure the stability of the material's magnetic properties, the ambient temperature cannot be greater than 500 °C in practice.

### 2.2 Dielectric Loss Mechanism

**Figure 1:**
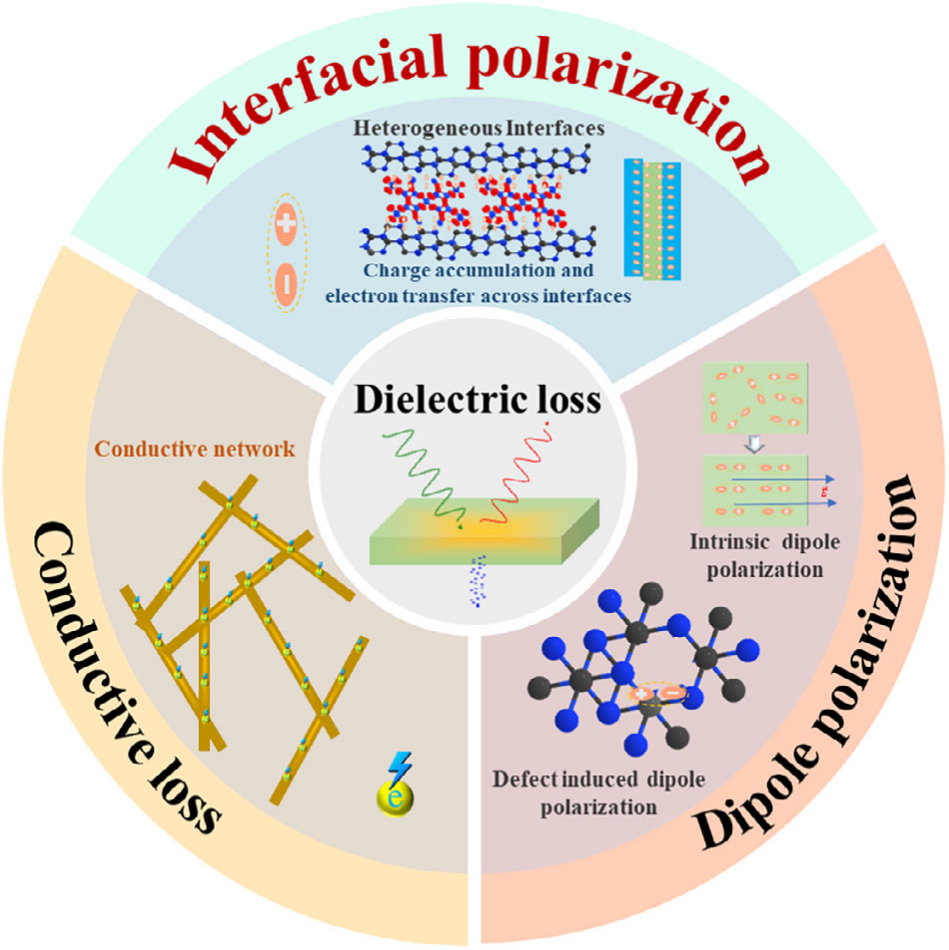
Schematic diagram of dielectric loss mechanism.

The dielectric loss mechanism plays an important role in high‐temperature environments. It can be categorized into three categories: conductivity loss, interfacial polarization loss, and dipole polarization loss, as illustrated in **Figure** **1**.^[^ ^37^, ^38^, ^39^ ^]^ In high‐temperature environment, the conditions triggered by these three loss mechanisms are different, and their relative contributions are significantly based on the material's composition and structure.^[^ ^40^, ^41^ ^]^

#### 2.2.1 Conductive Loss Mechanism

The conductive loss mechanism is that under an external alternating electromagnetic field, the internal charges of the material form a current in response to the electric field, generating Joule heat and dissipating electromagnetic wave energy.^[^ ^42^ ^]^ Through the design optimization of materials science such as building porous structures and developing composite materials’ systems, it can effectively promote the conduction efficiency of Joule heat, or achieve a balanced distribution of heat through structural innovation, thus alleviating the problem of thermal energy accumulation.^[^ ^43^, ^44^, ^45^, ^46^ ^]^ Conductivity loss is closely related to the conductivity of materials, such as carbon and conductive organics. However, excessively high conductivity can lead to impedance mismatch, resulting in excessive electromagnetic wave reflection on the material surface.^[^ ^47^ ^]^ At high temperature, electrons become extremely active, which leads to a substantial change in the conductivity of the material.^[^ ^48^ ^]^ This, in turn, causes a significant change in the overall dielectric constant of the material, thus attenuating the performance of the EMWAMs.^[^ ^49^ ^]^ Therefore, high conductivity is not recommended for high‐temperature EMWAMs. Constructing a microscopic conductive network in EMWAMs can effectively enhance their conductive loss. In addition, conductivity loss is significantly affected by temperature, because the essence of conductivity loss is the heat loss caused by the migration of electrons.

#### 2.2.2 Interfacial Polarization Loss Mechanism

Interfacial polarization losses typically manifest at heterogeneous interfaces, where significant differences in conductivity and dielectric properties exist, which causes the accumulation and redistribution of interfacial charges.^[^ ^50^, ^51^, ^52^, ^53^ ^]^ Due to the difference in conductivity and dielectric constant of the components on both sides, the obstacle of charge transfer through the interface becomes greater under the action of the external electric field, and the charge transport is easily restricted at the interface, thereby inducing interfacial polarization.^[^ ^54^, ^55^, ^56^ ^]^ Generally, interfacial polarization can be promoted from two aspects.^[^ ^57^ ^]^ One is to create multiple heterogeneous interfaces, and the other is to provide sufficient contact area between the absorbing material and the air medium.^[^ ^58^, ^59^, ^60^, ^61^ ^]^ Therefore, it is an effective method to prepare electromagnetic wave absorbing composites and porous EMWAMs to enhance the interfacial polarization loss.^[^ ^62^, ^63^, ^64^, ^65^ ^]^ For the EMWAMs in high‐temperature environments, the interface effect becomes more pronounced and active at a high temperature, this loss mechanism is particularly suitable.

#### 2.2.3 Dipole Polarization Loss Mechanism

Dipole polarization losses are divided into intrinsic dipolarization and defect‐induced dipolarization, with dipoles being generated by the displacement of positive and negative charges.^[^ ^66^, ^67^ ^]^ Under an external electromagnetic field, intrinsic dipole rearrangement will occur. While for nonpolar molecules, there is no intrinsic dipole. The principle of polarization relaxation loss is based on the difference in the electronegativity between two ions, causing electrons to flow and agglomerate to the more electronegative ion. This results in an uneven electron cloud distribution across the molecule or functional group at both ends to form an electric dipole.^[^ ^68^, ^69^, ^70^, ^71^ ^]^ It then polarizes and relaxes under an alternating electromagnetic field, consuming electromagnetic wave energy. The intrinsic dipolar polarization loss in EMWAMs stems from the presence of polar molecules and functional groups. The intrinsic dipolar polarization mainly comes from polar water molecules and functional groups.^[^ ^72^, ^73^, ^74^ ^]^ Thus, anchoring sufficient polar molecules and functional groups on the absorbing material can promote dipolar polarization loss. However, in high‐temperature environment, a large number of polar water molecules and oxygen‐containing functional groups are lost, resulting in the weakening of intrinsic dipole polarization. The functional groups in carbon‐based materials play a major role at high temperature. However, it is difficult for carbon‐based materials to retain the functional group. Defect‐induced polarization originates from the existence of defect sites in the EMWAMs. The principle of defect‐induced dipolarization is that there should be a certain charge around the defect sites in the material.^[^ ^75^, ^76^, ^77^, ^78^ ^]^ During the carrier transport and transfer process, the defect sites will trap the carriers opposite to the surrounding charges, resulting in uneven charge distribution and the formation of electric dipoles, resulting in electromagnetic wave energy losses.^[^ ^79^, ^80^, ^81^ ^]^ In addition, the oxygen vacancies introduced by thermal treatment, reduction process, cation doping, or heteroatom doping (e.g., F, N, P, and B) can also increase the defect sites in the absorber material, and thus boost the polarization loss.^[^ ^82^ ^]^ While at high temperature, because many doped atoms will precipitate, the original defects may change irregularly, rendering them difficult to manage.

The Cole–Cole circle and Debye polarization relaxation theory are adopted to judge the contribution of various loss mechanisms in EMWAMs at high temperature. In the Cole–Cole circle, a semicircle represents the occurrence of relaxation polarization, with the size of the circle representing the polarization intensity. The straight line represents the occurrence of conductivity loss, with the slope corresponding to the intensity of conductivity loss. According to Debye's polarization relaxation theory, the real and imaginary parts of the dielectric constant of complex media can be formulated as follows:^[^ ^83^ ^]^

(4) $$\varepsilon^{\prime }=\varepsilon_{\infty }+\frac{\varepsilon_{s}-\varepsilon_{\infty }}{1+{\left(2\pi f\right)}^{2}\tau^{2}}$$

(5) $$\varepsilon^{\prime \prime }={\varepsilon_{p}}^{\prime \prime }+{\varepsilon_{c}}^{\prime \prime }=\frac{\varepsilon_{s}-\varepsilon_{\infty }}{1+\omega^{2}\tau^{2}}\omega \tau +\frac{\sigma_{s}}{\omega \varepsilon_{0}}$$

where τ represents the polarization relaxation time, ε_s_ represents the static relative dielectric constant, *ε*_∞_ represents the high‐frequency limit relative dielectric constant, *ε*_p_" represents the imaginary part of the dielectric constant contributed by polarization loss, *ε*_c_" represents the imaginary part of the dielectric constant contributed by conductivity loss, *σ*_s_ represents the conductivity, and *ε*_0_ is the vacuum dielectric constant. Based on this, the conductivity loss decreases with increasing frequency. The proportion of contributions from polarization loss and dielectric loss can be calculated from Formulas (4) and (5) to regulate the loss effectively EMWAM at high temperature.

In the analysis of the electromagnetic loss mechanism, the method of combining the Cole–Cole arc analysis model with the Debye relaxation theory is adopted to systematically distinguish the contribution weights of dielectric loss and magnetic loss. Through the precise measurement of complex dielectric constant and magnetic permeability, the quantitative characterization of different loss mechanisms is realized.

## 3 The Electromagnetic Wave Absorption Testing Method of EMWAMs at High Temperature

The electromagnetic wave absorption performance testing methods for EMWAMs are mainly divided into the coaxial probe method (liquid measurement), transmission line method, free space method, resonant cavity method, parallel plate method, and inductance measurement method.^[^ ^84^ ^]^

The vector network analyzer is the core instrument and consists of a signal source, a receiver, and a display. The signal source emits a single‐frequency signal to the material under test. The receiver is tuned to this frequency and detects signals reflected and emitted by the material. The measured response is used to derive the amplitude and phase data at that frequency. Then, the source shifts to the subsequent frequency and repeats the above measurements to obtain frequency‐dependent reflection and transmission measurement responses.^[^ ^85^ ^]^ Owing to high temperature, numerous testing fixtures and samples are unsuitable for use. Consequently, the waveguide method is typically employed within the transmission line method, while within the free space method, the bow frame method is generally utilized, to test the performance of EMWAMs in high‐temperature environments. **Table** **1** systematically compares the high‐temperature testing methods of two electromagnetic wave absorbing materials.

**Table 1:** Comparison of different test methods.

	Waveguide method	Bow frame method
Mechanism	Determines electromagnetic parameters via *S*_11_ and *S*_12_ coefficients	Evaluates absorption properties by measuring reflectance at varying incident angles
Temperature range	Up to 1200 °C	Up to 1000 °C
Advantages	1. Small sample size 2. High accuracy 3. Fast testing	1. Noncontact testing 2. Support multiangle measurement 3. Broadband coverage
Disadvantages	1. Only one‐directional electromagnetic wave incidence 2. Need to change sample sizes frequently	1. Large sample area 2. Requires a large space darkroom
Applicable scenarios	Laboratory accurate measurements	Multiangle performance evaluation of large‐scale materials in practical applications

### 3.1 Waveguide Method

This method possesses the advantages of a small footprint, fast test speed, accurate results, and less sample consumption. It is one of the most prevalent methods for measuring microwave electromagnetic parameters at present.^[^ ^86^ ^]^ By measuring the reflection coefficient *S*_11_ and the transmission coefficient *S*_12_ of the sample transmission line section, the microwave electromagnetic parameters are determined by inverse calculation. The waveguide method requires a uniform sample composition, a smooth surface, and no gap with the fixture wall. The size of waveguide samples will vary depending on the measurement frequency, with the thickness ranging from 2 to 4 mm. The preparation method of samples also differs depending on the type of material. Solid materials need to be cut to the required size, while powder materials must be evenly mixed with paraffin wax at a certain filler ratio. A special mold is then used to form the required size. However, in high‐temperature environment, the preparation of powder samples will change because paraffin wax struggles to maintain its shape at high temperature. Other kinds of high‐temperature‐resistant wave‐transmitting materials are substituted and inorganic binders are employed to ensure the stability of sample shape in high‐temperature testing environment.

**Figure 2:**
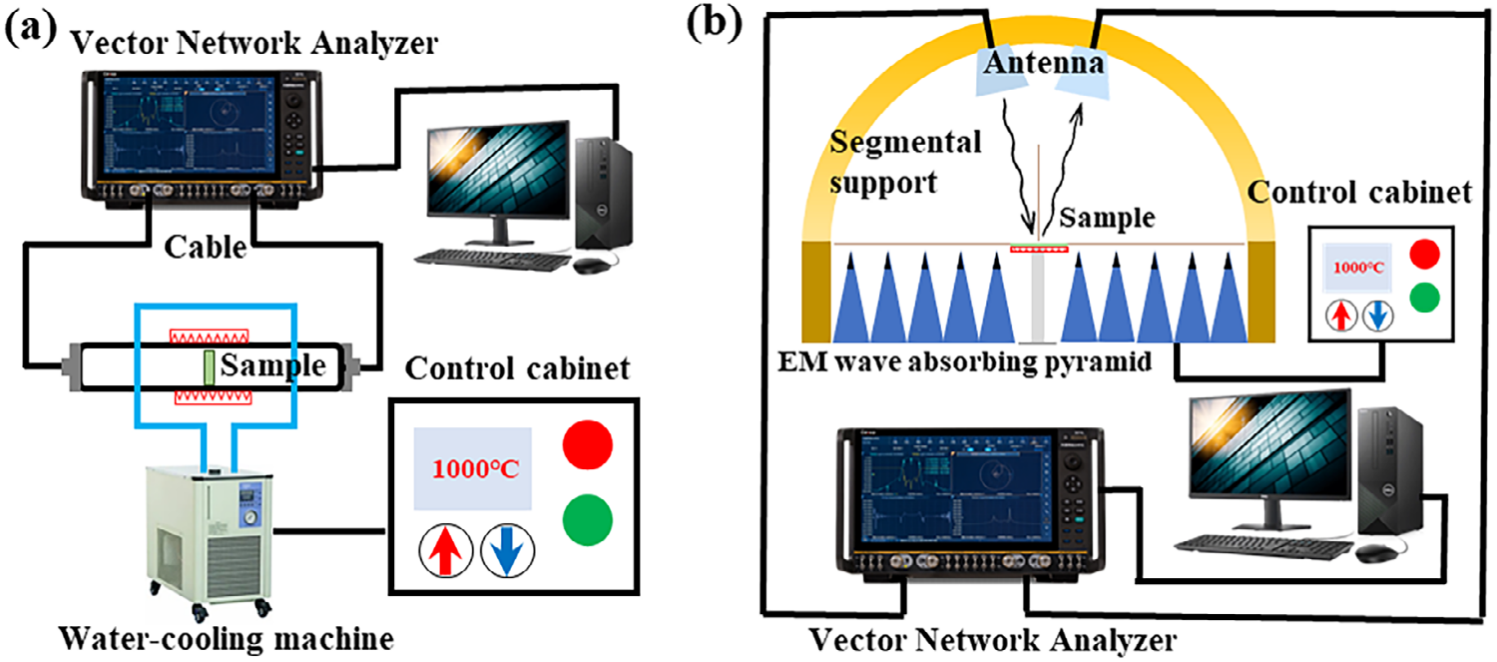
a) Schematic of the waveguide method test system at high temperature. b) Schematic of the bow frame method test system at high temperature.

A high‐temperature waveguide test equipment is shown in **Figure** **2**a. The device creates a high‐temperature environment by heating the area around the sample and cooling circulating water is used to reduce the temperature of the surrounding test components, ensuring smooth data transmission. By substituting the test fixture from ordinary metal to high‐temperature alloy or carbon fiber in the high‐temperature test environment, the device can operate at temperature as high as 800–1200 °C. Since the fixture materials tend to be oxidized, the high‐temperature test environment must be a vacuum, and this eliminates the interface polarization between the material and the air. To address this issue, the test environment atmosphere can be changed to argon. The test results have confirmed that the electromagnetic wave absorption performance measured in argon falls within an acceptable error margin compared to testing conducted in the air.

The test accuracy of the high‐temperature waveguide method is affected by such factors as the test temperature, sample dimensional accuracy, and test frequency. When the temperature exceeds 800 °C, the difference in thermal expansion coefficient between the sample and the fixture can easily cause deformation, resulting in fluctuation of the test curve. Therefore, it needs to strictly control the dimensional tolerance of the sample. At high temperature, the tiny gap will expand, leading to the reflection coefficient error. Because of the short electromagnetic wavelength at the high‐frequency band, it is more sensitive to the dimensional change of the sample.

To improve the accuracy, it is suggested to optimize fixture material compatibility by selecting thermally stable alloys or composites to minimize thermal expansion mismatch. At the same time, by adopting precision machining technology, the assembly gap is controlled below 50 µm, and the contact surface topography uniformity is ensured to reduce the nonideal fluctuation of interface contact resistance. On this basis, a repeatable experimental group (≥3 groups) was designed. Parallel experiments were performed strictly under the same environment and loading conditions. The mean value processing and error analysis of multiple groups of data were carried out by statistical methods, so as to effectively suppress the random errors caused by material microstructure heterogeneity or external environmental disturbance.

Nevertheless, this method also possesses certain limitations. For example, to obtain the performance data in a wide frequency range, it is necessary to prepare samples of various sizes and only the response of materials to electromagnetic waves in a specific direction can be tested. For multiangle and multidirection absorbing performance evaluation, this method is not applicable.

### 3.2 Bow Frame Method

Currently, the common test method used for EMWAMs studied in the literature is the transmission line method, while the waveguide method is the most commonly used for high‐temperature EMWAMs. The bow frame method is mainly used in military practical applications and is not used much in laboratories. Originally invented by the U.S. Naval Research Laboratory in the late 1940s, this method serves as a stealth material testing technology widely adopted in the military. As its name implies, the signal‐transmitting wire and the signal‐receiving wire are installed on the semicircular shelf above the EMWAM plate to be measured, and the template is placed at the center position of the bow frame, as shown in Figure 2b.^[^ ^87^ ^]^ The reflectance of the EMWAMs at different incident angles can be tested by changing the position of the antenna on the bow frame. The ability of the absorbing material to absorb electromagnetic waves is evaluated by the reflectance with the reflectivity unit of decibels (dB). This measurement method is the most intuitive, rather than through calculations. The sample needs to be coated or sprayed on a metal plate of 18 × 18 cm or 30 × 30 cm, or it can be placed directly on the metal plate. The sample's thickness should be less than 5 mm and the surface of the sample should be flat and uniform. The test frequency is 1–40 GHz. Because the test is noncontact, it will not cause damage to the material. The incident angle can be adjusted to test the influence of material anisotropy on electromagnetic wave absorption ability, and the test frequency range is wide. There is a heating plate under the sample plate, with an insulating cover plate placed atop it. After the ambient temperature around the absorbing material reaches a high temperature by heating the heating plate, the cover plate is removed for testing, allowing for test temperatures as high as 1000 °C. However, this method also has its shortcomings. First, the sample should possess a certain strength. Second, since the coating technology is adopted, the bonding force between the EMWAMs and the metal base plate and the cracking of the coating should also be considered. For block samples, to avoid the problem of fracture caused by insufficient strength, multiblock splicing is adopted to obtain the required area. Lastly, this test method demands a considerable area and necessitates a large dark room.

The test accuracy of the bow frame method mainly depends on the following factors including the control accuracy of incident angle and reflection angle, and the effective area of the plate sample. During the test, it is necessary to ensure that the angle error between the incident direction of the electromagnetic wave and the normal line of the sample surface is less than ±1° to accurately capture the anisotropic response of the material. To avoid environmental reflection interference, broadband absorbing materials are usually laid around the periphery of the sample, and time domain gate technology is used to filter stray signals in the nonsample area. The sample size is inversely proportional to the test frequency. In practical application, the sample size needs to be dynamically optimized according to the target frequency band.

At high temperature, the activity and mutual influence of molecules within materials intensify. However, exploring microscopic means at such temperatures poses greater challenges. Therefore, EMWAMs at high temperature remain application‐oriented, focusing on lightweight materials, and have high mechanical strength and good electromagnetic wave absorption performance, in line with the needs of national equipment. In the future, microscopic characterization of the properties of EMWAMs can be carried out using techniques such as off‐axis electron holography, in situ X‐ray diffraction (XRD), in situ X‐ray photoelectron spectroscopy (XPS), in situ Raman spectroscopy, in situ transmission electron microscopy (TEM), in situ Hall effect, in situ conductivity, electron pin resonance/electron paramagnetic resonance (ESR/EPR), X‐ray absorption spectroscopy (XAS), positron annihilation spectroscopy (PAS), ultraviolet‐visiblediffuse reflectance spectroscopy (UV‐Vis‐DRS), and aberration‐corrected TEM (AC‐TEM). Advanced characterization techniques can be used to detect the changes in microscopic properties, facilitating semiquantitative detection of these factors and their correlation with control measures.^[^ ^88^ ^]^ By relating changes in microscopic properties to macroscopic parameters, e.g., matching thickness, absorption bandwidth, matching frequency, and peak loss, the relationship between microscopic properties and electromagnetic wave absorption mechanisms can be explored.

## 4 EMWAMs at High Temperature

EMWAMs at high temperature can be divided into two major categories, i.e., magnetic absorber and dielectric absorber. The loss capacity of magnetic absorbing material results from the joint action of magnetic loss and dielectric loss mechanism. It is crucial to optimize the magnetic permeability and dielectric constant for optimal impedance matching. However, the magnetic permeability of magnetic materials undergoes significant change with temperature increases, leading to impedance mismatch and a substantial decrease in electromagnetic wave absorption capacity.^[^ ^89^ ^]^ Consequently, the application of magnetic materials as high‐temperature absorbing material should be below their Curie temperature.^[^ ^90^, ^91^, ^92^, ^93^, ^94^ ^]^

There are still some studies on the application of magnetic materials in the field of high‐temperature electromagnetic wave absorbing materials. Wang et al. successfully prepared high‐temperature microwave absorbers with magnetic metals as additives.^[^ ^91^ ^]^ The Co@C/Na_2_SiO_3_ composites demonstrated stability at 400 °C, and the evaluation of the static magnetic properties revealed their excellent antioxidant properties in high‐temperature environments. These properties remained magnetic at this temperature. At 400 °C, the X‐band could be obtained at a thickness of 1.7 mm with −8 dB. Wang et al. prepared a high‐temperature wave absorbing material (Nd_2_Co_17_@C/Na_2_SiO_3_) based on recent rare‐earth soft magnetic alloys (Nd_2_Co_17_) that exhibit high permeability and a Curie temperature.^[^ ^90^ ^]^ The composite was stabilized at 450 °C following carbon coating and Na₂SiO_3_ treatment. The reflection loss intensity of the composite in the entire X‐band is less than −6 dB at a thickness of 1.5 mm. It can be seen that the application of magnetic materials is mainly concentrated below 500 °C.

In high‐temperature environments, the choice of electromagnetic wave absorbing materials is significantly limited. Since only pure dielectric material systems can be selected, its impedance matching characteristics make it difficult to achieve a good balance, and performance improvement often comes at the expense of increasing material thickness. Thus high‐temperature wave absorbing materials usually require a larger thickness. To achieve the best impedance matching of thinner thickness under these conditions, it is necessary to introduce multiple loss mechanisms to enhance the dielectric properties of the materials to make up for the problem of weak intrinsic dielectric properties of ceramic matrix materials.

**Figure 3:**
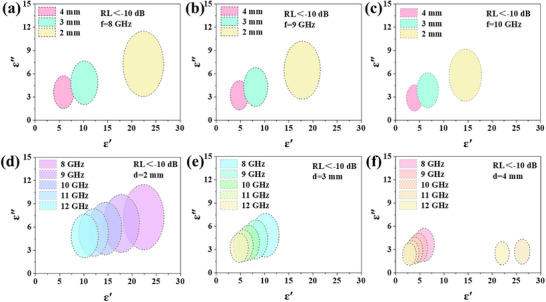
The permittivity corresponding to RL below −10 dB when the RL as a function of real part and imaginary part of permittivity for the dielectric materials: a) 8 GHz, b) 9 GHz, and c) 10 GHz at different thicknesses, and d) 2 mm, e) 3 mm, and f) 4 mm at different frequencies.

According to Formula (2), the real part of the fixed permeability of the nonmagnetic dielectric material is 1 and the imaginary part is 0. RL is a quantity related to the three parameters *f*, *d*, and *ε*_r_, which implies that under a given frequency and material's thickness, the corresponding reflection loss RL value can be adjusted by modifying the real and imaginary parts of the dielectric constant *ε*_r_. Similarly, to obtain materials with RL ←10 dB, the required dielectric constant *ε* “and imaginary parts *ε*” should be designed. As the frequency increases, the required dielectric constant range decreases and shifts toward the low dielectric constant range, as shown in **Figure** **3**. As the thickness decreases, the required dielectric constant range expands and moves toward the high dielectric constant range, which aligns with the impedance matching condition given by Formula (1). This provides a direction for the subsequent work of improving the absorbing performance through the regulation of the dielectric constant.

### 4.1 Carbon‐Based Materials

#### 4.1.1 Graphite

**Figure 4:**
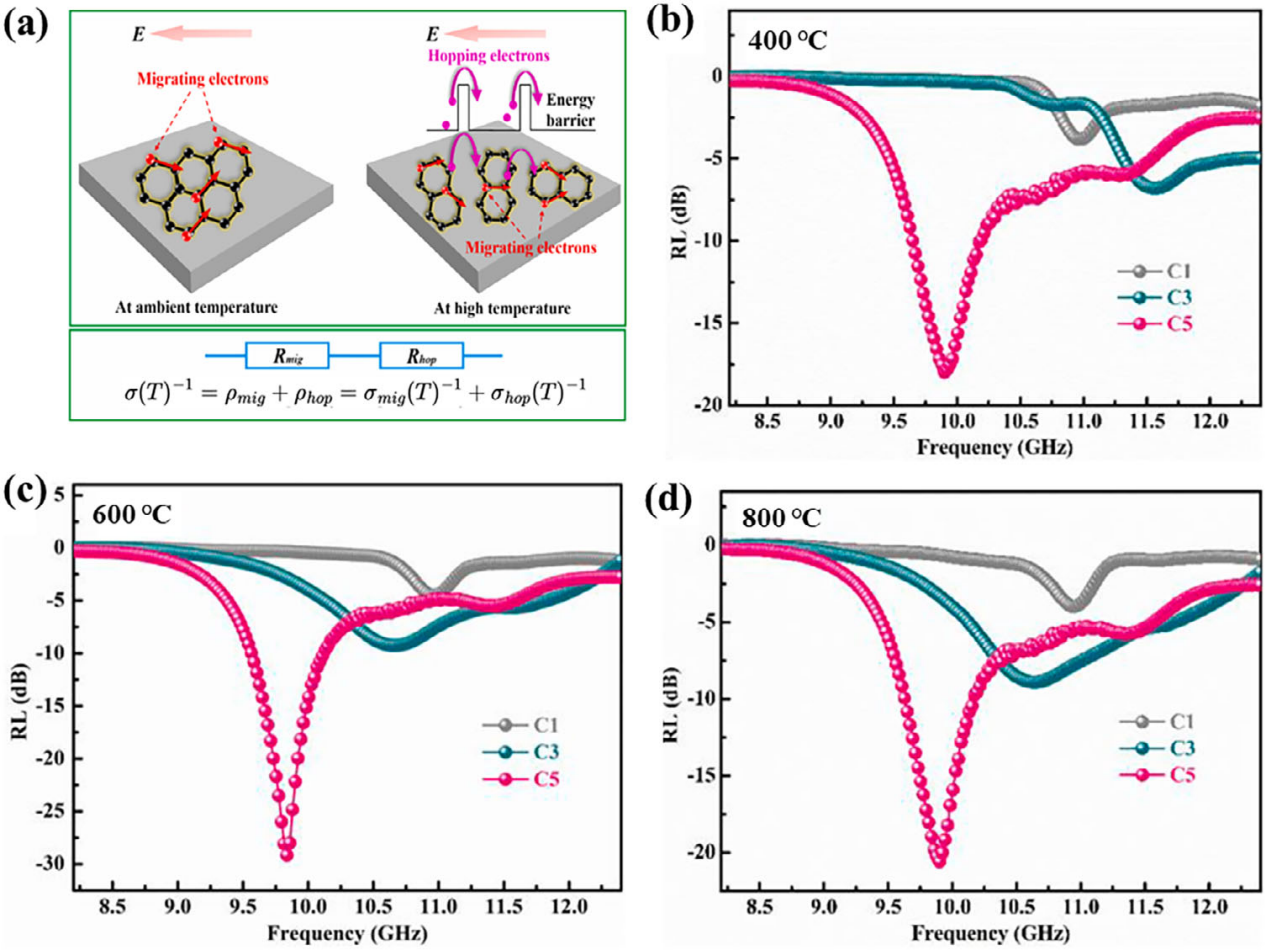
a) Electronic conduction mechanism of SiBCN ceramics. b–d) RL curves at high temperature different graphite content (C1–C5 increase in turn) in SiBCN ceramics. Reproduced with permission.^[^ ^100^ ^]^ Copyright 2022, Elsevier.

Graphite plays an important role in the field of EMWAMs because of its excellent electrical conductivity, chemical stability, and high temperature resistance, and is especially suitable for scenarios with high‐temperature, high‐strength, and low‐density requirements. However, the dielectric thermal stability of graphite is inadequate. With the increase in temperature, the dielectric constant of graphite will increase rapidly, resulting in impedance mismatch. Consequently, it is not advisable for use in scenarios involving a wide range of temperatures.^[^ ^95^, ^96^, ^97^, ^98^, ^99^ ^]^ Li and co‐workers prepared SiBCN ceramics by adjusting the boron content. At a thickness of 2.0 mm, the ceramic exhibited a minimum reflection loss of −35.25 dB at 10.57 GHz. SiBCN ceramics exhibited excellent electromagnetic wave attenuation performance at 600 °C.^[^ ^100^ ^]^ Specifically, for a ceramic thickness of 2.5 mm, the minimum reflection loss reached −29.18 dB at 9.65 GHz. The reason is that the increase in boron promotes the transformation of amorphous carbon to crystalline graphite, increasing the heterogeneous interface and conductive pathways, as shown in **Figure** **4**. The absorbing property of the material increases with temperature due to the enhancement of electromigration and electron jumping in the graphite layer, but decreases at 800 °C due to the partial oxidation of graphite at high temperature, suggesting that the material should not be utilized at temperatures greater than 600 °C. Observing the thermogravimetric curve of the material, it becomes evident that the material's weight declines rapidly above 600 °C.

#### 4.1.2 Carbon Fiber

**Figure 5:**
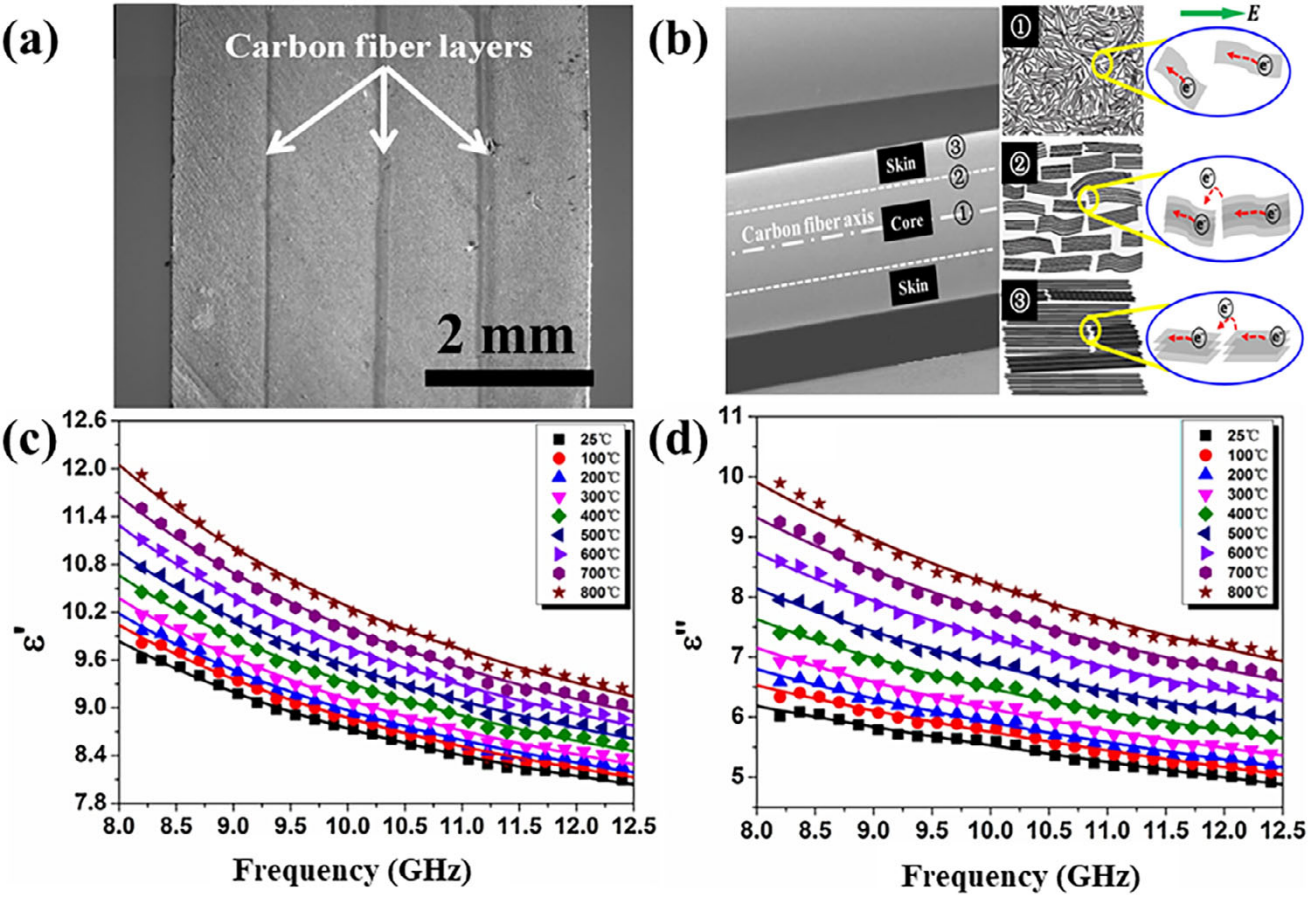
a) Image of the cross section of multilayer C_f_/Si_3_N_4_ composites. b) Sketches of microstructure arrangement and electronic motion in carbon fiber. c) The real part permittivity for C_f_/Si_3_N_4_ composites in X‐band at high temperature. d) The imaginary part permittivity for C_f_/Si_3_N_4_ composites in X‐band at high temperature. Reproduced with permission.^[^ ^106^ ^]^ Copyright 2017, Elsevier.

Carbon fiber has become a kind of excellent EMWAMs due to its excellent conductivity, high strength, and chemical stability.^[^ ^101^, ^102^ ^]^ It is usually compounded with other materials to further optimize its absorbing ability.^[^ ^103^, ^104^, ^105^ ^]^ Peng and co‐workers fabricated C_f_/Si_3_N_4_ composites through a layer‐stacking process, and Si_3_N_4_ ceramic slurry was prepared by ball‐milling Si_3_N_4_ powder with Al_2_O_3_ and Y_2_O_3_ sintering additives. Subsequently, carbon fibers were evenly distributed onto the slurry surface and cured using an initiator–catalyst system. This fiber‐layering and curing procedure was repeated twice to construct a three‐layer structure. Finally, the stacked preform underwent drying followed by oxygen‐free sintering to achieve the final composite material.^[^ ^106^ ^]^ The composite exhibits excellent dielectric properties, and the dielectric constant and loss tangent change little with the change of temperature, which is mainly attributed to the enhancement of electric polarization and relaxation of electron migration in graphitic basal planes of short carbon fibers, as shown in **Figure** **5**. These characteristics are very conducive to the absorption of electromagnetic waves in a wide temperature range. Unfortunately, from the imaginary and real parts of the dielectric constant of this material, it does not exhibit good electromagnetic‐wave‐absorption performance.

#### 4.1.3 Carbon Nanotubes

**Figure 6:**
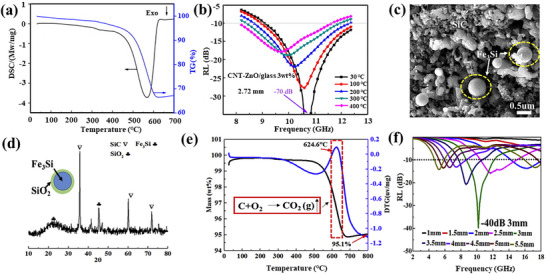
a) Thermogravimetry‐differential scanning calorimetry (TG–DSC) curves of CNT–ZnO powder in air atmosphere. b) RL curves of CNT–ZnO/glass composite with 3 wt% filler loading at high temperature. Reproduced with permission.^[^ ^109^ ^]^ Copyright 2015, Elsevier. c) Scanning electron microscopy (SEM) image, d) XRD patterns, e) derivative thermogravimetry‐thermogravimetry (DTG–TG) curves, and f) RL curves of SiC/Fe_3_Si/CNTs composite after oxidation in air at 800 °C. Reproduced with permission.^[^ ^110^ ^]^ Copyright 2019, Elsevier.

Carbon nanotubes are commonly used as absorbing material due to their low density, high electrical conductivity, large specific surface area, and unique 2D structure.^[^ ^107^, ^108^ ^]^ Yin and co‐workers prepared carbon nanotube–ZnO composite powder as high‐temperature electromagnetic wave absorber by a homogeneous method and then prepared carbon nanotube–ZnO/glass composite by a pressureless sintering method.^[^ ^109^ ^]^ The minimum reflection loss of this material can reach −22 dB at 400 °C, with an absorption bandwidth of 3.2 GHz. The good performance is attributed to the assembly of ZnO nanoparticles within carbon nanotubes, which produces heterostructures and enhances the polarization of heterointerfaces, as shown in **Figure** **6**a,b. Due to the consumption of amorphous carbon layers on the outer surface of the carbon nanotubes and the generation of oxygen vacancies in ZnO during sintering, it causes more relaxation polarization and dielectric loss in the electromagnetic field. Wen and co‐workers prepared a novel SiC/Fe_3_Si/carbon nanotubes (CNTs) composite using polymer‐derived ceramics with a minimum RL value of −41.2 dB at 10.5 GHz at a thickness of 2 mm and effective bandwidth of close to 4 GHz (12.9–16.9 GHz) at a thickness of only 1.5 mm.^[^ ^110^ ^]^ After oxidation in air at 800 °C, the absorbent maintained its main structure and showed good resistance to high‐temperature oxidation. The absorber still exhibits excellent absorbing properties with a minimum RL value of −40 dB at a thickness of 3 mm and a bandwidth of 4.8 GHz (10.4–15.2 GHz) at a thickness of 2.5 mm, as shown in Figure 6c–f. Thermogravimetric analysis shows that the carbon‐containing part will be oxidized under air conditions after 600 °C, losing its role in adjusting impedance matching. However, in this paper, due to the small amount of carbon and the overall structure of the material after carbon depletion, the dielectric properties and impedance matching of the material still maintain good electromagnetic wave absorption capacity at high temperature.

#### 4.1.4 Graphene

**Figure 7:**
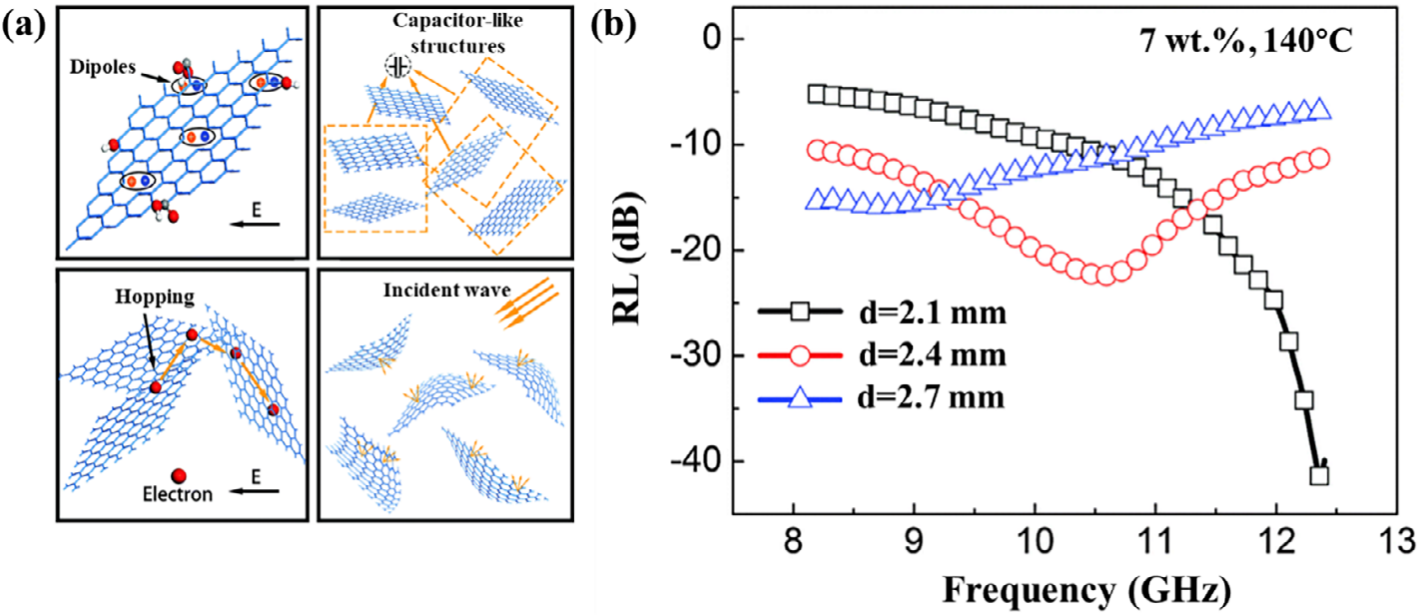
a) Schematic diagram of the mechanism of electromagnetic wave absorption of the ultrathin graphene composite. b) RL curves of the 7 wt% ultrathin graphene composite at 140 °C. Reproduced with permission.^[^ ^113^ ^]^ Copyright 2015, Pubs.

Graphene is often used in EMWAMs because of its ultrathin, ultralight nature, and strong conductivity.^[^ ^111^, ^112^ ^]^ For high‐temperature EMWAMs, the dielectric constant of graphene does not change significantly with the increase in temperature, which is highly beneficial for wide temperature domain absorption. Cao et al. obtained graphene oxide by vigorously oxidizing commercial graphite powder and obtained ultrathin graphene by reducing graphene oxide with the hydroiodic acid and acetic acid solution, followed by freeze drying.^[^ ^113^ ^]^ The graphene was mixed and pressed with silica xerogel powder to make a composite material, which exhibited efficient electromagnetic wave absorption and a thermally stable dielectric constant, with a minimum reflection loss of −42 dB and the widest bandwidth covering the entire X‐band, as shown in **Figure** **7**. Unfortunately, the test temperature is only maintained at 140 °C, which may be due to the limitations of the test equipment or test methods.

**Figure 8:**
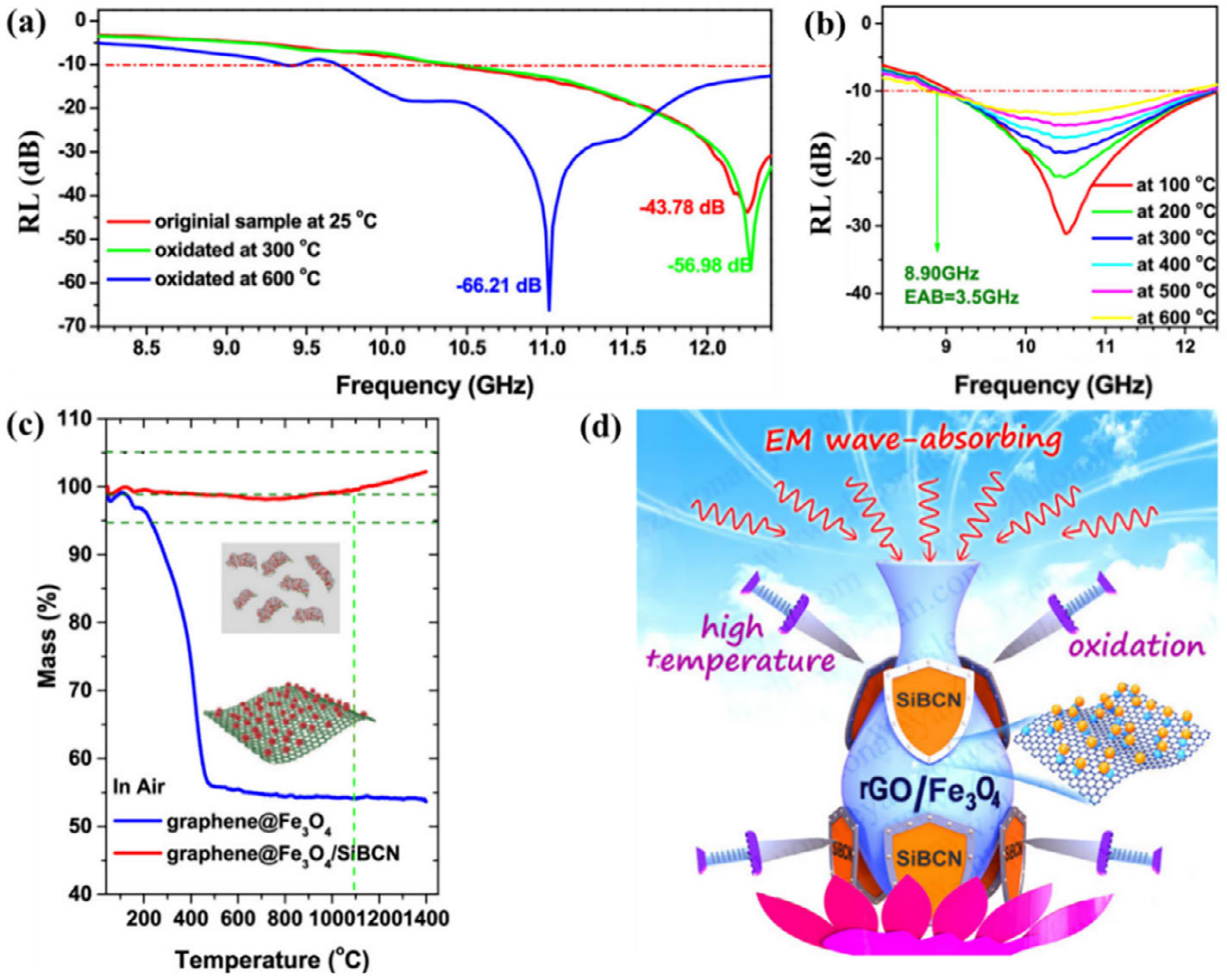
a) RL curves of graphene@Fe_3_O_4_/SiBCN complexes at 25 °C and after oxidation at 300 and 600 °C for 2 h in air. b) RL curves of graphene@Fe_3_O_4_/SiBCN complexes at high temperature. c) TG curves of graphene@Fe_3_O_4_ and graphene@Fe_3_O_4_/SiBCN complex in air atmosphere. d) Schematic diagram of graphene@Fe_3_O_4_/SiBCN complexes with resistance to high‐temperature oxidation. Reproduced with permission.^[^ ^114^ ^]^ Copyright 2018, ACS.

Kong and co‐workers pointed out that the Achilles’ heel of carbon materials is that it is easily oxidized in high temperature and oxidizing conditions, especially at temperatures above 600 °C.^[^ ^114^ ^]^ To address this, graphene@Fe_3_O_4_/SiBCN nanocomposites with an A/B/C structure were designed, in which SiBCN served as a “shield” to protect graphene@Fe_3_O_4_ from oxidation, as shown in **Figure** **8**. The results revealed that, despite this protection, the carbon in the material undergoes gradual consumption after 2 h at 600 °C, leading to a decrease in its dielectric constant. Since the dielectric constant is sensitive to temperature, it is challenging to utilize these materials across a wide temperature range.^[^ ^115^, ^116^, ^117^, ^118^ ^]^ In addition, there is Fe_3_O_4_ in this material, and the influence of magnetic permeability on the absorbing performance has yet to be conducted.

Carbon‐based materials are unstable at high temperatures. When the ambient temperature exceeds 600 °C, the material surface will undergo a violent oxidation reaction with atmospheric oxygen to generate CO_2_. The oxidation kinetics process shows an obvious temperature dependence, with the reaction rate increasing exponentially as the temperature rises. This high‐temperature instability is in contrast to ceramic materials, which can still maintain excellent chemical inertness and structural integrity in high‐temperature environments due to the synergistic effect of strong covalent bonds and ionic bonds.

Carbon‐based materials will cause intensified lattice vibration under thermal excitation, resulting in a significant increase in carrier mobility. This makes its conductivity increase in order of magnitude with the increase in temperature, leading to the abnormal increase of the imaginary part of the dielectric constant. The impedance matching characteristics of carbon‐based materials deteriorate once the temperature exceeds the critical threshold, leading to an increase in electromagnetic wave reflection and failure of the loss mechanism, which ultimately causes a sharp decline in absorption performance.

### 4.2 Ceramic‐Based Materials

In the research of high‐temperature EMWAMs, the extreme environment exceeding 800 °C poses stringent demands on the stability and absorbing performance of materials, constituting a significant challenge for high‐temperature EMWAMs. From the preceding summary, it can be seen that the maximum operating temperature of carbon‐based materials is limited to 600 °C. Exceeding this temperature leads to oxidation in a high‐temperature aerobic environment, significantly compromising the absorption performance of the entire microwave absorbing composite. Carbon‐based materials demonstrate prominent applications in high‐temperature electromagnetic protection systems. A key implementation lies in their integration into supersonic aircraft fuselage surfaces, where they serve as electromagnetic shielding layers operating stably at ≈300 °C. Beyond aerospace, these materials are extensively utilized in industrial high‐temperature equipment, such as temperature measurement windows for metallurgical furnaces and protective casings for high‐temperature sensors. These applications not only fulfill stringent electromagnetic compatibility requirements but also ensure robust material stability under extreme thermal conditions. Presently, ceramic materials, particularly SiC ceramics, stand out as the primary candidates capable of sustaining their properties over extended durations in high‐temperature conditions.^[^ ^115^, ^116^, ^117^, ^118^ ^]^ SiC ceramic material has garnered significant attention as a research focal point in high‐temperature EMWAMs due to its cost‐effectiveness and exceptional dielectric thermal stability. Its robust thermal and chemical stability empowers it to retain superior electromagnetic wave absorption capacity in high‐temperature settings and play an irreplaceable role as aerospace hot‐end components, such as engine nozzles and turbine blades where the temperature exceeds 800 °C.

#### 4.2.1 Doped SiC

**Figure 9:**
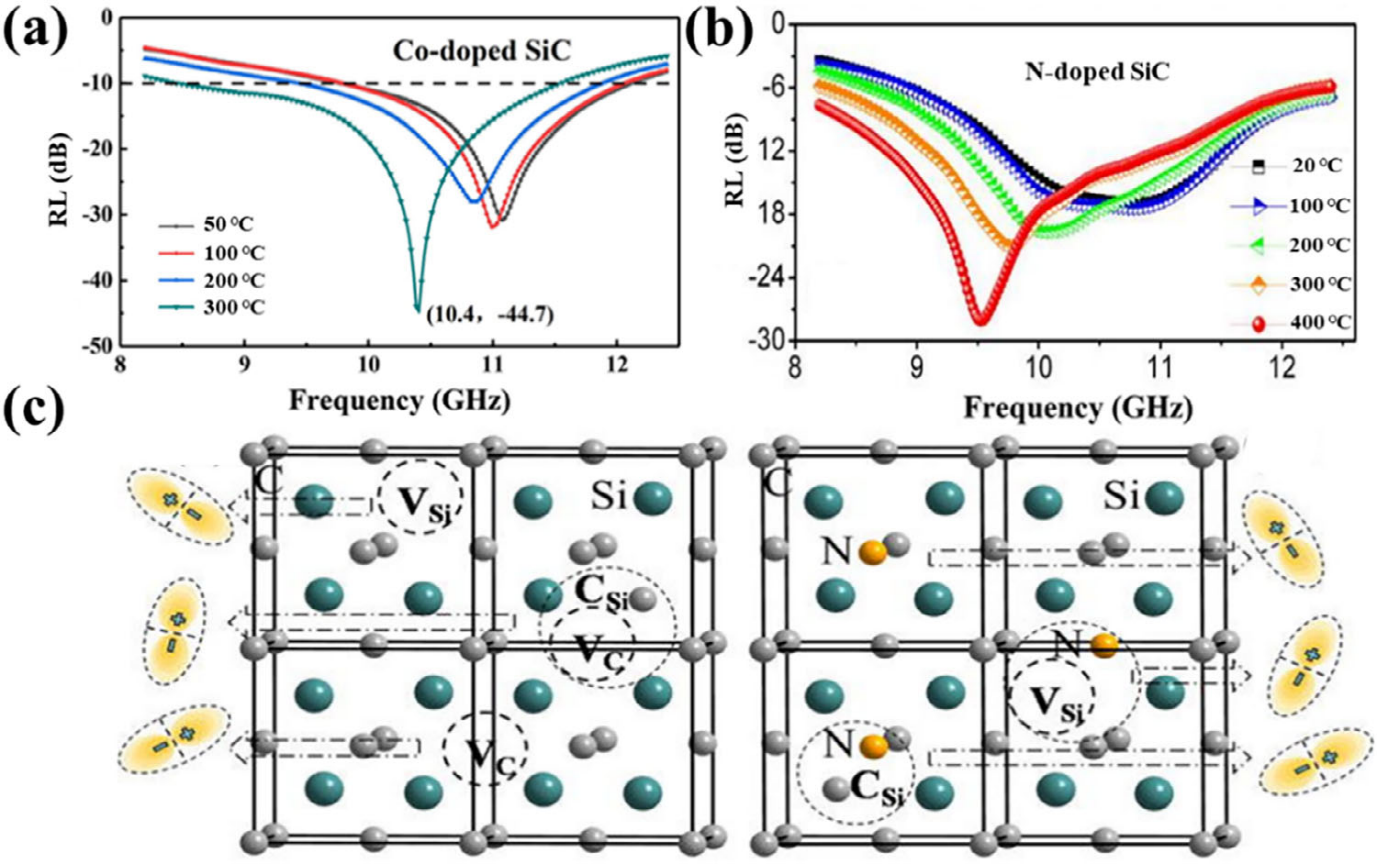
a) RL curves of Co‐doped SiC at high temperature. Reproduced with permission.^[^ ^137^ ^]^ Copyright 2018, Elsevier. b) RL curves of N‐doped SiC at high temperature. c) Electromagnetic wave absorbing mechanism of N‐doped SiC. Reproduced with permission.^[^ ^138^ ^]^ Copyright 2014, Pubs.

SiC is a highly suitable material for high‐temperature electromagnetic wave absorption, owing to its exceptionally high melting point, remarkable thermal stability, superior dielectric properties, and outstanding mechanical strength.^[^ ^119^, ^120^, ^121^, ^122^, ^123^, ^124^, ^125^, ^126^, ^127^, ^128^, ^129^, ^130^ ^]^ As an absorbing material, doped SiC has attracted much attention in recent years primarily due to the introduction of doping elements that significantly improve its electromagnetic wave absorption performance.^[^ ^131^, ^132^, ^133^ ^]^ Doped SiC materials retain high structural stability and absorbing properties at high temperatures. Different doping elements and doping concentrations can flexibly adjust the electromagnetic properties of materials, allowing their performance to be optimized and designed according to specific needs.^[^ ^134^, ^135^, ^136^ ^]^ As reported by Jin and co‐workers, nanostructured co‐doped SiC powders with core/shell heterogeneous phases were synthesized by the method of mechanical activation assisted combustion.^[^ ^137^ ^]^ Co‐doped SiC exhibits good absorbing performance in the X‐frequency band. At an extremely small thickness of 1.7 mm, the RL_min_ value of Co‐doped SiC reaches −44.7 dB, which is >3 times that of undoped SiC, as shown in **Figure** **9**a. The introduction of Co significantly improves the electromagnetic wave absorption of the material, especially at high temperatures. The excellent electromagnetic wave absorption performance of Co‐doped SiC can be attributed to two factors: the abundance of active sites due to the enhanced polarization loss of defect dipole and the enhancement of conductivity due to more conductive carriers with increasing temperature. The foregoing examples mainly attribute the enhanced dielectric losses and conductivity observed at high temperatures to the polarization of the defects. This polarization leads to an increase in the number of active sites, which in turn improves the electrical conductivity of the material. Therefore, the dielectric constant increases with temperature, thereby achieving excellent impedance matching. This ultimately leads to an improvement in electromagnetic wave absorption at high temperatures. As shown in Figure 9b,c, Cao and co‐workers prepared N‐doped SiC materials, wherein N doping was found to significantly enhance the electromagnetic wave absorption capacity of SiC.^[^ ^138^ ^]^ The minimum reflection loss of N‐doped SiC was observed to reach ≈−30 dB at 400 °C, with an effective absorption bandwidth of 3 GHz. The exceptional high‐temperature dielectric properties are attributed to the presence of multiple relaxation processes induced by N‐doping and vacancy defects resulting from dipole polarization relaxation.

#### 4.2.2 SiC Nanowires

**Figure 10:**
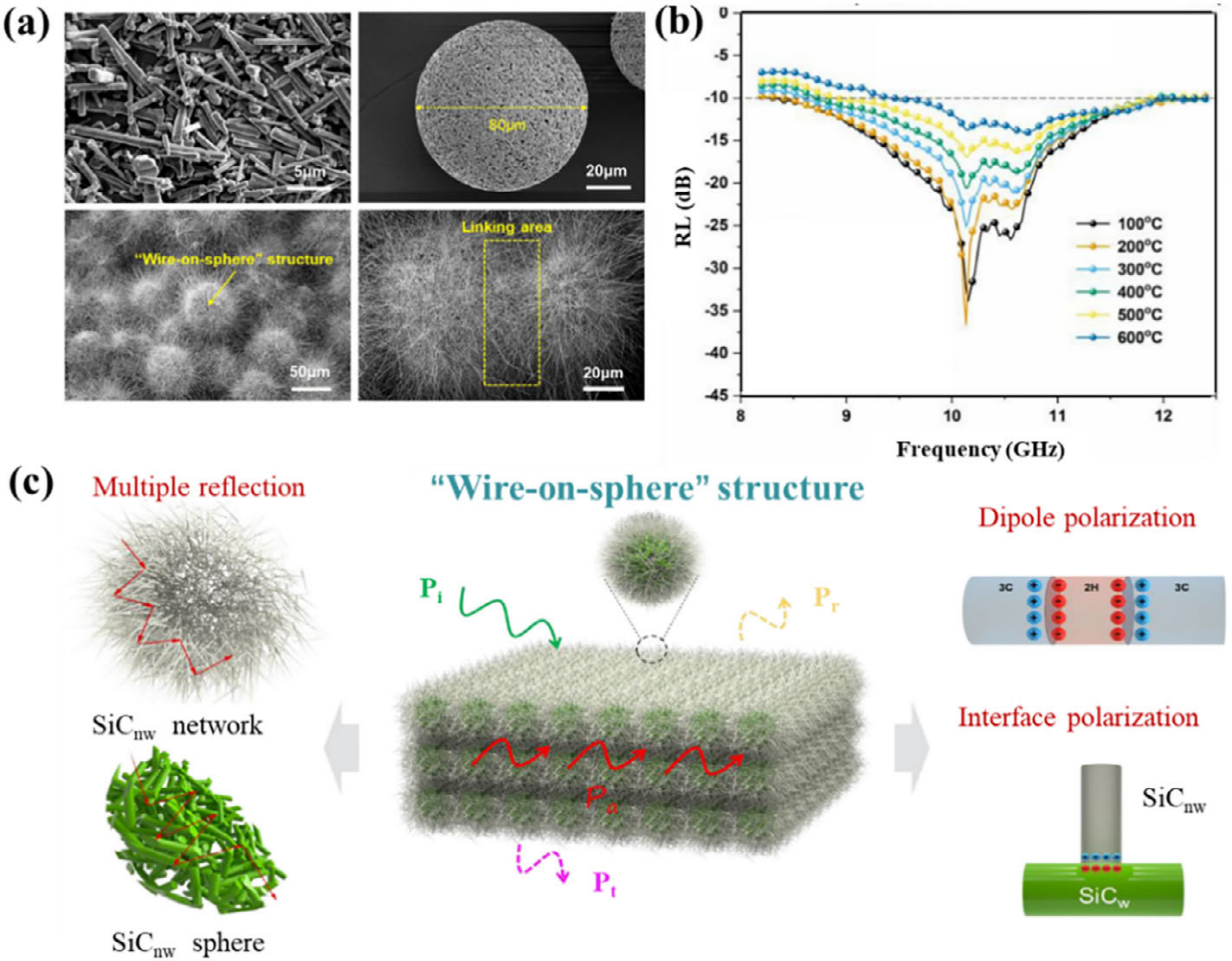
a) SEM images of SiC_nw_/SiC_w_ foam. b) RL curves of SiC_nw_/SiC_w_ foam at high temperature. c) Schematic diagram of the mechanism of electromagnetic wave absorption of SiC_nw_/SiC_w_ foam. Reproduced with permission.^[^ ^139^ ^]^ Copyright 2022, Elsevier.

Ye and co‐workers developed SiC nanowires/SiC whiskers (SiC_nw_/SiC_w_) foams with a unique “ball‐on‐wire” hierarchical structure by assembling SiC_w_ onto porous SiC_w_ spheres spray‐drying followed by 3D printing of SiC_w_ spheres onto SiC_w_ foam.^[^ ^139^ ^]^ At room temperature, the maximum electromagnetic wave effective absorption bandwidth (EAB_max_) and the minimum electromagnetic wave RL_min_ of SiC_nw_/SiC_w_ foam can reach 4 GHz and −57 dB, respectively. At 600 °C, the EAB_max_ and RL_min_ were 3 GHz and −15 dB, respectively, as shown in **Figure** **10**.

**Figure 11:**
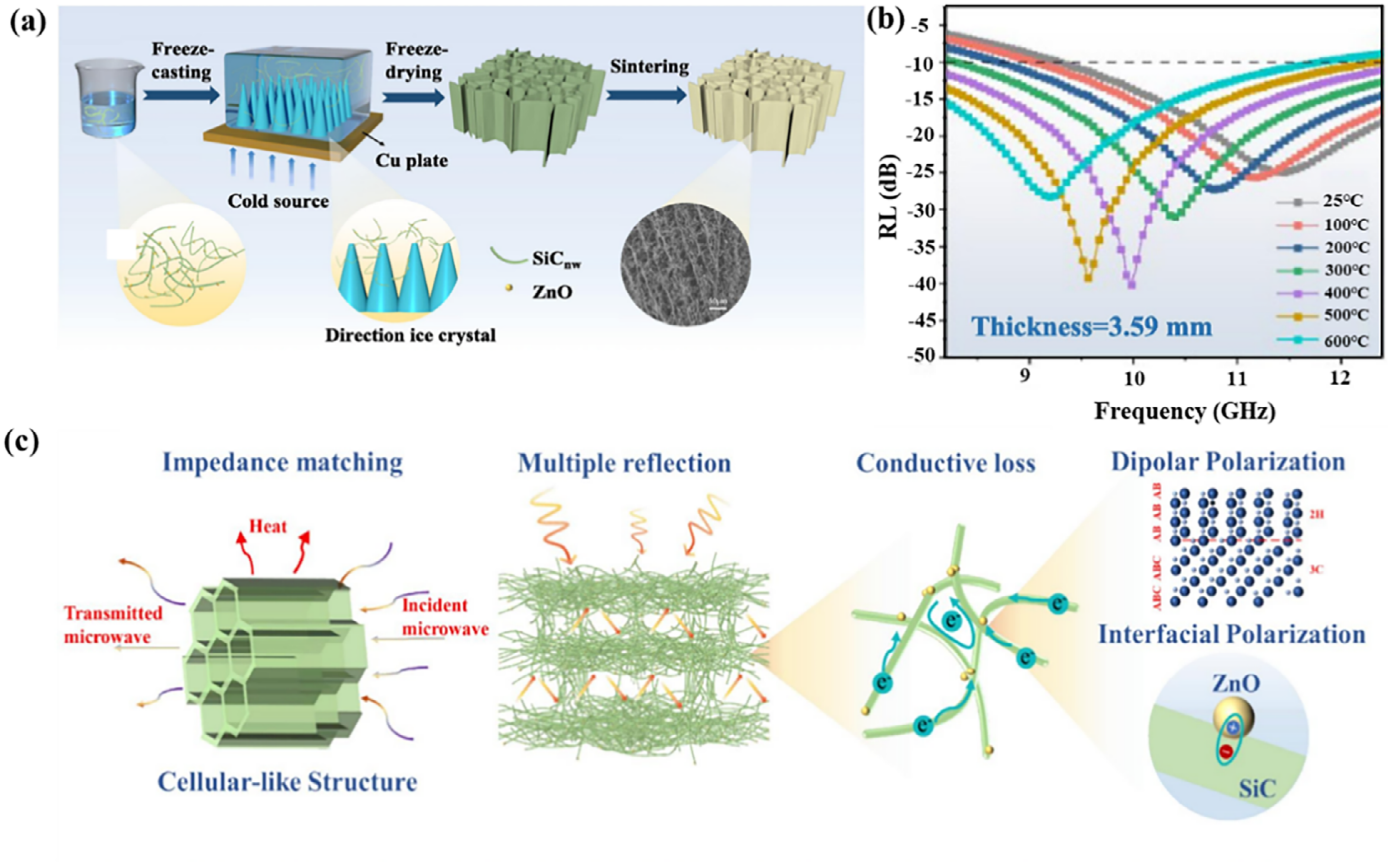
a) Schematic diagram of the preparation process of ZnO/SiC_nw_ aerogel. b) RL curves of ZnO/SiC_nw_ aerogel at high temperature. c) Schematic diagram of the mechanism of electromagnetic wave absorption of ZnO/SiC_nw_ aerogel. Reproduced with permission.^[^ ^140^ ^]^ Copyright 2024, Elsevier.

With their extremely low density, aerogels are ideal EMWAMs for use in aviation, aerospace, and other weight‐sensitive industries. Their micro–nano structure can significantly increase the probability of multiple reflections and scattering of electromagnetic waves, thereby enhancing their electromagnetic wave absorption capabilities. Zhu and co‐workers designed a laminated porous structure aerogel material to grow ZnO on SiC_nw_ by dual hydrolysis reaction method and then prepared ZnO/SiC_nw_ aerogel by the directional solidification method.^[^ ^140^ ^]^ The material shows excellent electromagnetic wave absorption performance at room temperature to 600 °C and effective broadband attenuation (>90%) in the entire X‐band, as shown in **Figure** **11**.

**Figure 12:**
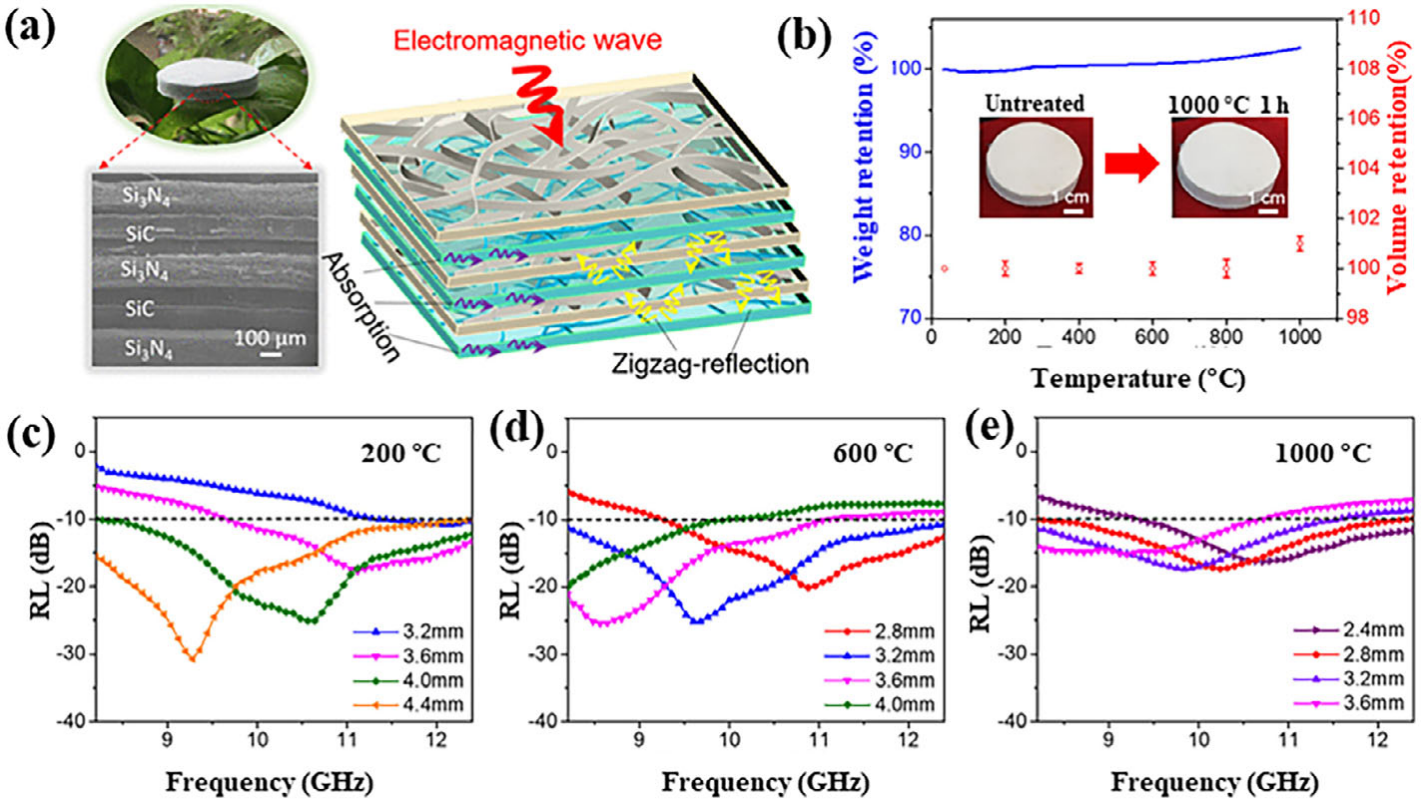
a) SEM image and schematic diagram of the mechanism of electromagnetic wave absorption of multilayer SiC/Si_3_N_4_ ceramic aerogel. b) TG curves and thermal stability of the multilayer SiC/Si_3_N_4_ ceramic aerogel. c–e) RL curves of multilayer ceramic aerogel SiC/Si_3_N_4_ at high temperature. Reproduced with permission.^[^ ^141^ ^]^ Copyright 2021, ACS.

Su and co‐workers prepared a high‐performance electromagnetic wave absorbing ceramic aerogel composed of multilayer wave‐transmitting Si_3_N_4_ layers and wave absorbing SiC layers alternately constructed by a simple composite method.^[^ ^141^ ^]^ The prepared aerogels possess ultralow density (≈8 mg cm^−3^), wide effective absorption bandwidth (8.4 GHz), strong reflection loss at room temperature (−45 dB), and excellent electromagnetic wave absorption performance at high temperatures up to 1000 °C, as shown in **Figure** **12**. The good electromagnetic wave absorption performance is attributed to the well‐designed alternating multilayer structure, which causes the reflection–absorption–zigzag reflection process significantly enhancing the loss of electromagnetic waves. However, the underlying loss mechanism and polarization mechanism are not clear. At high temperature, the conductivity loss of materials is an important mechanism of electromagnetic wave absorption. To improve the conductivity loss of materials by constructing more conductive paths, and then enhance their wave absorbing ability, in this direction, adopting 1D SiC (such as SiC nanowires or fibers) structures has significant advantages, because 1D structures can provide more conductive pathways and promote the conversion and dissipation of electromagnetic wave energy.

#### 4.2.3 SiC Derivatives

**Figure 13:**
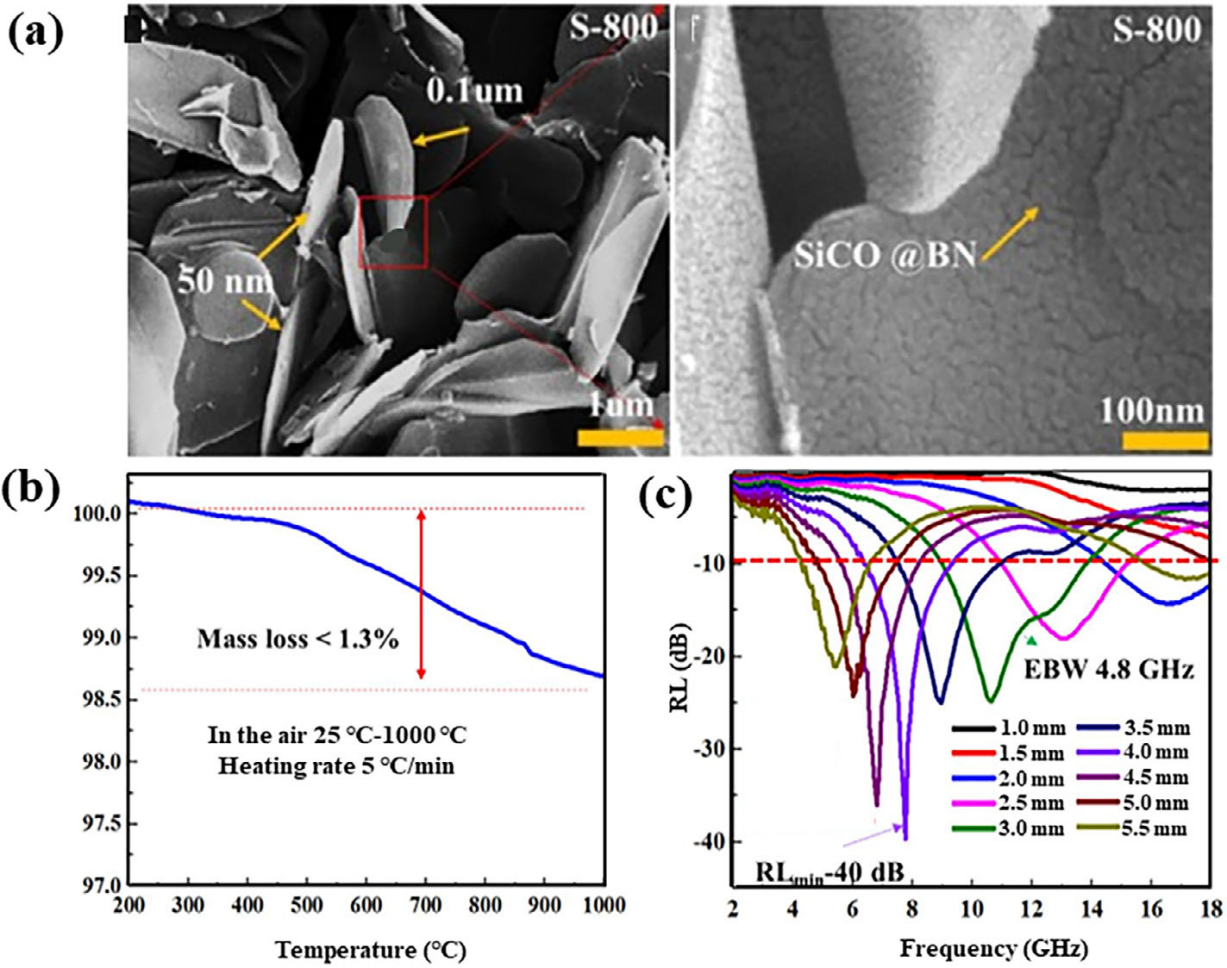
a) Morphology, b) TG curve, and c) RL curves of SiCO@BN composite. Reproduced with permission.^[^ ^142^ ^]^ Copyright 2019, Elsevier.

SiC‐derived ceramics such as SiCN, SiCO, and SiBCN can be used as absorbents by adjusting the element content and selecting a suitable pyrolysis temperature. Huang and co‐workers coated polymer‐derived SiCO ceramics on 2D BN sheets, and the resulting materials exhibited excellent wave absorbing properties.^[^ ^142^ ^]^ At a thickness of 4 mm, the calculated minimum reflection loss reaches −55.6 dB at 8.7 GHz, and the widest effective bandwidth approaches 5.9 GHz (12–17.9 GHz) at a thickness of 2.5 mm. Moreover, the SiCO@BN composite has good oxidation resistance at high temperatures, with a mass loss of <1.3% even after heating in air at 1000 °C. The associated RL_min_ and EBW were still −40 dB at a thickness of 4 mm and 4.8 GHz (8.9–13.7 GHz) at a thickness of 3 mm, as shown in **Figure** **13**. Since the material in question is a pure ceramic, the mass loss incurred after heating in the air is minimal, and it is foreseeable that its electromagnetic wave absorption performance at high temperatures will also be exceptional. Nonetheless, due to limitations imposed by the testing instrumentation, it is challenging to measure the high‐temperature dielectric parameters of powder materials. Interfacial polarization can generate local polarization within materials due to differing dielectric properties, thereby enhancing the absorption of electromagnetic waves.

Under high‐temperature conditions, one of the pivotal strategies to boost the absorbing capacity is to augment the interfacial polarization effect by constructing heterogeneous interfaces. Our research team has undertaken numerous attempts to address this challenge, making strides in several experiments and obtaining preliminary results through the establishment of effective heterogeneous interfaces and the enhancement of interfacial polarization losses. These endeavors have furnished invaluable experience for further refining the properties of high‐temperature absorbing materials.

#### 4.2.4 Titanium Carbide

**Figure 14:**
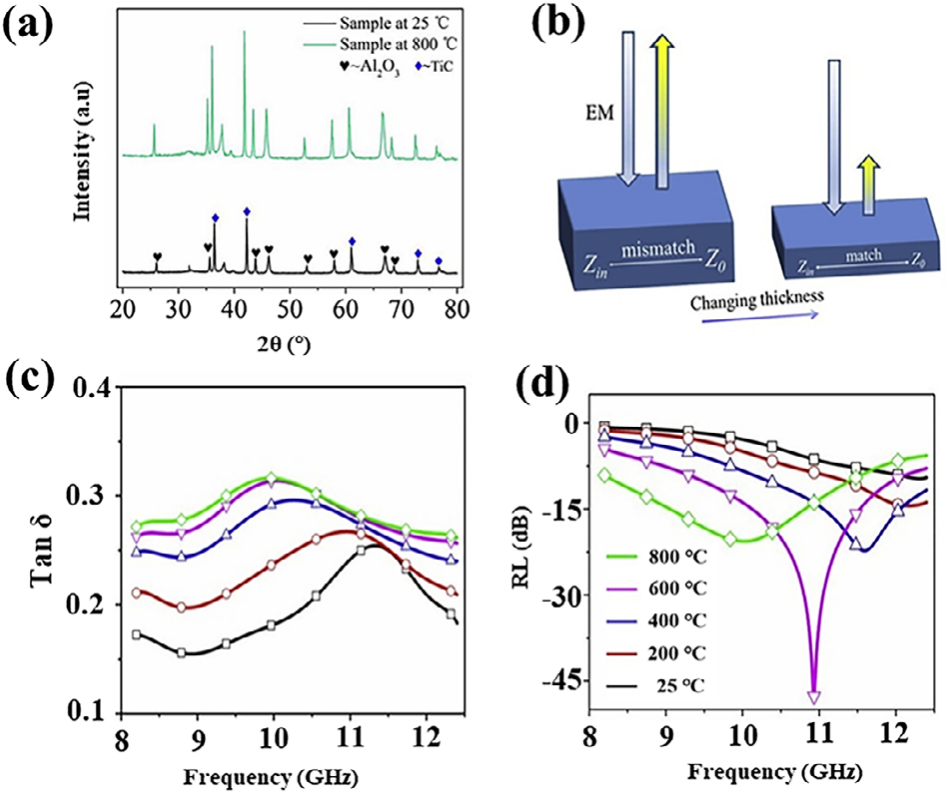
a) XRD pattern of the TiC content of 20% coating at 25 and 800 °C. b) Diagram of input impedance for thickness change. c) Loss factors and d) RL curves of the TiC content of 20% coating at high temperatures in the X‐band. Reproduced with permission.^[^ ^146^ ^]^ Copyright 2020, Elsevier.

Ceramic‐based EMWAMs encompass not only SiC but also other types such as AlN, TiC, and BaTiO_3_. These materials possess the characteristics of high‐temperature resistance and chemical stability. However, they pose challenges in shape design and precision control.^[^ ^143^, ^144^, ^145^ ^]^ Ma and co‐workers prepared the base powder by adjusting the ratio of Al_2_O_3_ and TiC and sprayed the absorbing material onto the metal plate by plasma‐spraying technology.^[^ ^146^ ^]^ The dielectric constant of the coating increases with the increase in temperature and TiC content. When the content of TiC is 20% and the thickness of the coating is 1.6 mm, the EAB is 3.45 GHz. The coating retains excellent electromagnetic wave absorption properties even at 800 °C. At this temperature, the reflection loss over the entire X‐band is below −8 dB. The XRD results showed that only two phases were present in the coating. The dielectric constant and impedance matching of the coating are controlled by the content of TiC, resulting in conductivity loss and interfacial polarization due to the inhomogeneous dispersion of titanium carbide in alumina, as shown in **Figure** **14**. However, this coating material also has obvious shortcomings, such as large reflection loss and a narrow applicable temperature range. This is attributed to TiC's sensitivity to temperature changes in its dielectric constant, and it is easy to cause impedance mismatch with the change in temperature. Additionally, the material system's simplicity and lack of diverse loss mechanisms present areas for improvement.

#### 4.2.5 MAX Phase Ceramics

**Figure 15:**
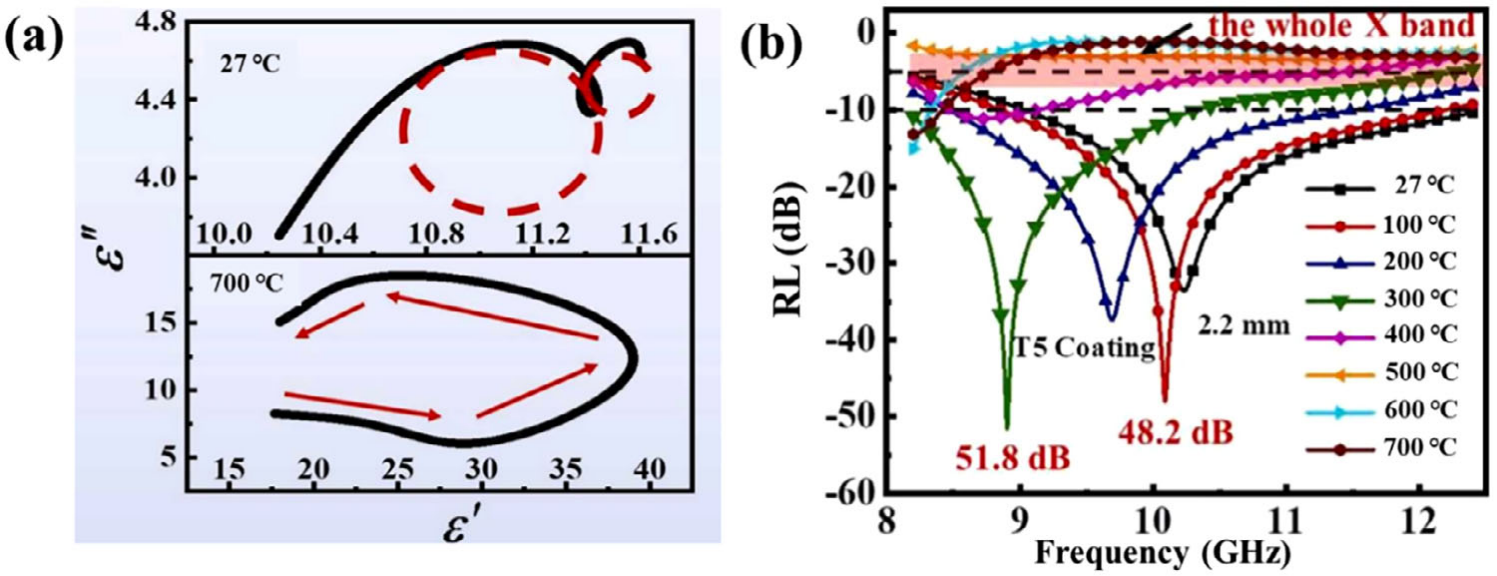
a) Cole–Cole plots of complex permittivity for *T*5 coating at 27 and 700 °C. b) RL of *T*5 coatings with 2.2 mm thicknesses across different temperatures. Reproduced with permission.^[^ ^147^ ^]^ Copyright 2024, Elsevier.

The MAX phase exhibits considerable high‐temperature microwave absorption potential due to its relatively high melting point and electrical conductivity. Wang et al. prepared Ti_3_SiC_2_/Al_2_O_3_‐13% TiO_2_ hetero coatings by plasma‐spraying technique.^[^ ^147^ ^]^ With 5 wt% Ti_3_SiC_2_ content, the coating achieved an effective bandwidth of 2.2 GHz at 600 °C with a thickness of only 1.4 mm. The loss mechanism is mainly that the increase of Ti_3_SiC_2_ content leads to the increase of polarization components, which improves the sensitivity to dielectric dispersion. As temperature increases, polarization within the coating gradually establishes, prompting the transition from polarization relaxation loss to conduction loss as the main dielectric loss mechanism, as shown in **Figure** **15**. Zhang and co‐workers successfully prepared Al_3_BC_3_ powder with purity up to 98.8 wt% using laser induced self‐propagation synthesis method from aluminum powder, boron carbide powder, and carbon powder as raw materials.^[^ ^148^ ^]^ The powder exhibits excellent EMW absorption performance at 700 °C, with reflection loss below −10 dB in the frequency range of 6.74–7.97 GHz and minimum reflection loss reaching −45.86 dB at a thickness of 2.9 mm.

Carbon‐based materials benefit from high plasticity and low‐temperature processing, enabling cost‐effective fabrication of complex geometries. Therefore, they excel in lightweight, flexible designs for supersonic aircraft surfaces below 600 °C, and industrial shielding. The electromagnetic loss mechanism of carbon‐based materials mainly relies on conductive loss and interfacial polarization to achieve broadband absorption below 600 °C. However, their conductive networks are highly sensitive to temperature, resulting in large conductivity changes at high temperatures and in turn impedance mismatch. What is more, carbon‐based materials undergo rapid oxidation in aerobic environments above 600 °C, resulting in reduced dielectric constant and reduced absorption properties.

In contrast, ceramic‐based materials dominate ultrahigh temperature scenarios (>800 °C), providing unparalleled stability for engine nozzles and turbine blades. The electromagnetic loss mechanism of ceramic‐based materials mostly adopts two loss mechanisms, i.e., defect‐induced polarization and nonuniform interfacial polarization. Among them, the interfacial polarization mechanism can remain stable under extreme thermal conditions, and dielectric losses can be achieved even above 800 °C. Due to the strong covalent/ionic bonds, ceramic‐based materials can be used as an indispensable part of aerospace hot‐end components. However, ceramic‐based systems require energy‐intensive processes. They incur higher equipment costs and energy consumption.

### 4.3 Metamaterial‐Based Materials

Since the magnetic loss mechanism is ineffective at higher temperatures, the objectives of “thin,” “light,” “wide,” and “strong” of high‐temperature EMWAMs solely through the dielectric loss mechanism alone is exceedingly difficult. Furthermore, the suitable composite materials are also quite limited.

**Figure 16:**
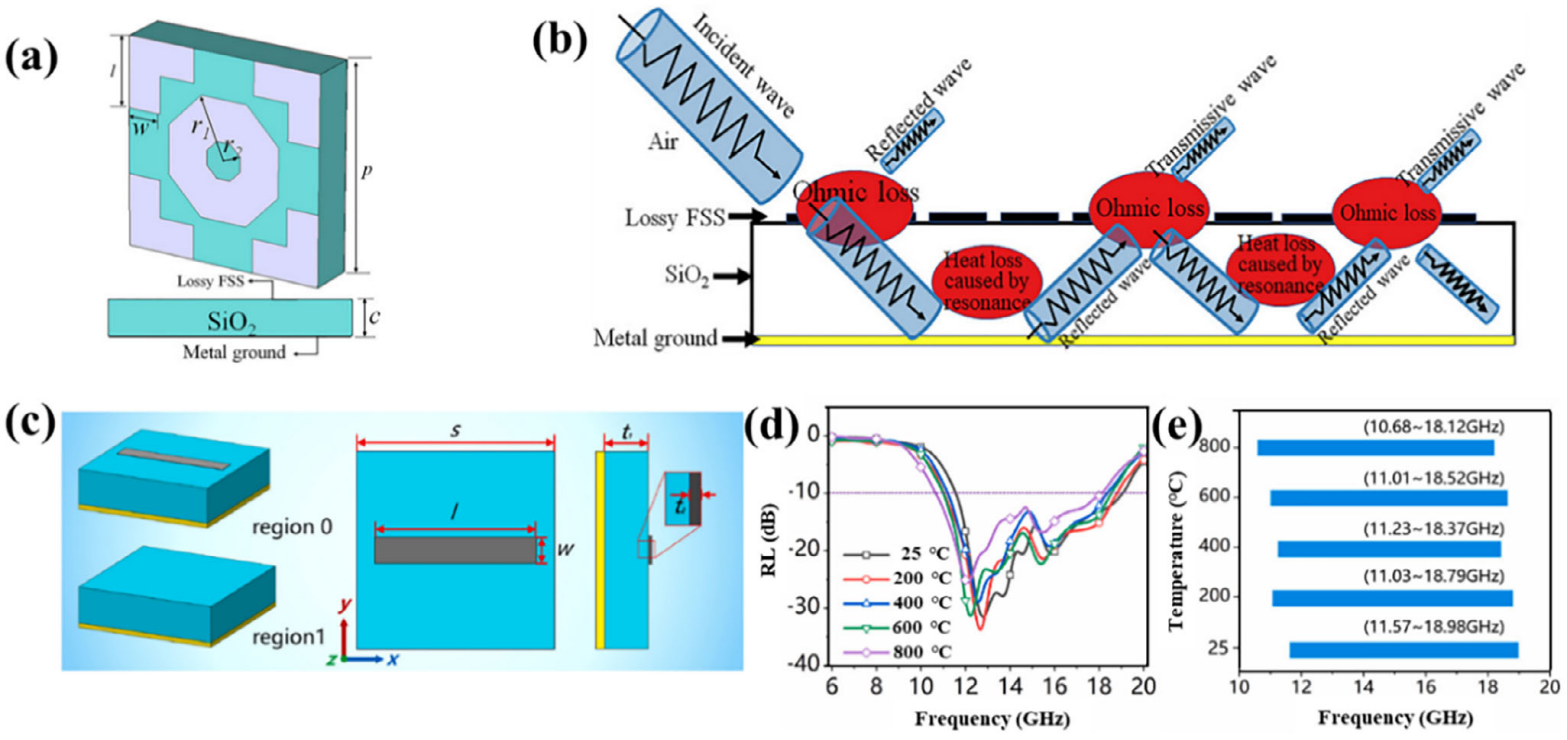
a) Diagram of the high‐temperature‐resistant broadband metamaterial absorber with a regular octagonal annular cross structure. b) Schematic diagram of the mechanism of electromagnetic wave absorption. Reproduced with permission.^[^ ^169^ ^]^ Copyright 2022, Wiley. c) Schematic of the Yb_2_SiO_5_ unit structure. d) RL curves of the Yb_2_SiO_5_ ceramic coating. e) Comparison of the frequency bandwidth when RL is below −10 dB at high temperature. Reproduced with permission.^[^ ^170^ ^]^ Copyright 2021, Elsevier.

To tackle these challenges, a significant number of researchers have augmented the absorbing performance of materials through the creation of metamaterial structures.^[^ ^95^, ^149^, ^150^, ^151^, ^152^ ^]^ With their special adjustment mechanism, metamaterials can shift the absorption frequency to lower frequencies and increase the absorption bandwidth, thereby making up for the shortcomings caused by the pure dielectric loss mechanism.^[^ ^122^, ^153^, ^154^, ^155^, ^156^, ^157^, ^158^, ^159^ ^]^ In addition, metamaterials can also reduce the amount of materials to meet the requirements of lightweight and improve the mechanical properties of materials, which is of great value in practical applications.^[^ ^160^, ^161^, ^162^, ^163^, ^164^, ^165^, ^166^, ^167^, ^168^ ^]^ Li and co‐workers designed and fabricated a high‐temperature‐resistant broadband metamaterial absorber using an orthogonal octagonal ring cross structure as a frequency‐selective surface as shown in **Figure** **16**a–c.^[^ ^169^ ^]^ The raw material is high‐temperature conductive paste, and the middle layer is SiO_2_. The results show that the reflection loss of the absorber is less than −5 dB at room temperature in the normal incidence range from 5.17 to 18 GHz. At 800 °C, the absorption bandwidth extends to 13.3 GHz (4.72–18 GHz). A comprehensive analysis shows that the main electromagnetic wave absorption mechanisms are conductance loss and resonance loss. Ma and co‐workers proposed homogeneous multifunctional materials and artificial design methods to design ultrathin metasurfaces, as shown in Figure 16d,e.^[^ ^170^ ^]^ Two materials, Yb_2_SiO_5_ and NiCrAlY, were selected as substrates and conductive units, and prototypes with staggered patterns were fabricated using atmospheric pressure plasma spraying. Simulation and experimental results show that the radar cross‐section (RCS) decreases significantly over a wide frequency range and exhibits stable decreasing performance over a wide temperature range.

**Figure 17:**
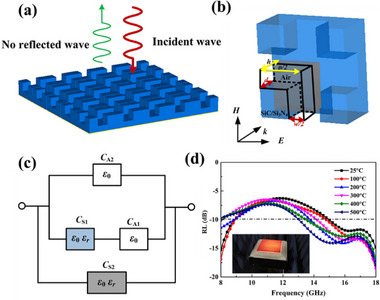
a) The schematic of the crossing grooved metastructure with SiC_f_/Si_3_N_4_ composites. b) Schematic of structural parameters of SiC_f_/Si_3_N_4_ composite metamaterial. c) The synthesized capacitance model of SiC_f_/Si_3_N_4_ composite metamaterial unit structure. d) RL curves of the crossing grooved structure SiC_f_/Si_3_N_4_ composites at high temperature. Reproduced with permission.^[^ ^171^ ^]^ Copyright 2018, Wiley.

Zhang and co‐workers proposed a multiscale design method to design a special high‐temperature, low–high‐frequency cooperative EMWAM by combining the microstructure and metastructure of the material, as shown in **Figure** **17**.^[^ ^171^ ^]^ SiC_f_/Si_3_N_4_ composites were prepared by chemical vapor infiltration with SiC_f_ as electromagnetic wave absorption phase and Si_3_N_4_ as a transparent ceramic matrix. To ensure the stability of absorbing ability at high temperature, a cross‐slotted element structure is designed and fabricated. Minimum reflection losses of −15.3 and −14.8 dB were observed at 8 and 18 GHz, respectively, with a total thickness of 5 mm. The reflection loss of the designed metastructure maintains a relatively reliable high‐temperature electromagnetic wave absorption performance in the range from room temperature to 500 °C. This effectively enhanced electromagnetic wave absorption performance is believed to be the result of multiscale effects generated by the combination of conventional EMWAMs with metamaterial structures.

From **Table** **2**, the important parameters of the different materials analyzed were compared. At the temperature range of 400–600 °C, carbon‐based materials have become the mainstream of high‐temperature EMWAMs due to their excellent electrical conductivity and high‐temperature resistance. However, it is prone to oxidation in an oxidation environment exceeding 600 °C. Above 800 °C, ceramic materials are ideal for their excellent high‐temperature stability and dielectric properties, among which SiC‐based materials are the most promising candidates. However, materials, solely on the dielectric loss mechanism, result in high absorption frequency and large thickness. Metamaterials, as an emerging material system, have attracted more and more attention because of their unique array structure, which effectively increases the width of absorption frequency.

**Table 2:** Comparison of key parameters of different materials.

Material type	Material	Maximum operating temperature [°C]	Optimum absorption properties	Refs.
Carbon‐based materials	Graphite‐doped SiBCN ceramics	600	RL_min_ = −35.25 dB EAB_max_ = 0.5 GHz	[100]
	Carbon nanotube‐ZnO/glass composite	400	RL_min_ = −22 dB EAB_max_ = 3.2 GHz	[109]
	SiC/Fe_3_Si/CNTs composite	800	RL_min_ = −40 dB EAB_max_ = 4.8 GHz	[110]
	Graphene/SiO_2_ composite	140	RL_min_ = −42 dB EAB_max_ = 4 GHz	[113]
	Graphene@ Fe_3_O_4_/SiBCN complexes	600	RL_min_ = −66.21 dB EAB_max_ = 3.5 GHz	[114]
Ceramic‐based materials	N‐doped SiC	400	RL_min_ = −30 dB EAB_max_ = 3 GHz	[138]
	SiC_nw_/SiC_w_ foams	600	RL_min_ = −57 dB EAB_max_ = 4 GHz	[139]
	ZnO/SiC_nw_ aerogel	600	RL_min_ = −40 dB EAB_max_ = 4 GHz	[140]
	SiC/Si_3_N_4_ ceramic aerogel	1000	RL_min_ = −45 dB EAB_max_ = 8.4 GHz	[141]
	SiCO@BN composite	1000	RL_min_ = −55.6 dB EAB_max_ = 5.9 GHz	[142]
	TiC/Al_2_O_3_ coating	800	RL_min_ = −46 dB EAB_max_ = −3.5 GHz	[146]
	Ti_3_SiC_2_/Al_2_O_3_‐TiO_2_ coating	700	RL_min_ = −51.8 dB EAB_max_ = 4 GHz	[147]
	Yb_2_SiO_5_ ceramic coating	800	RL_min_ = −33 dB EAB_max_ = 13.3 GHz	[170]

**Figure 18:**
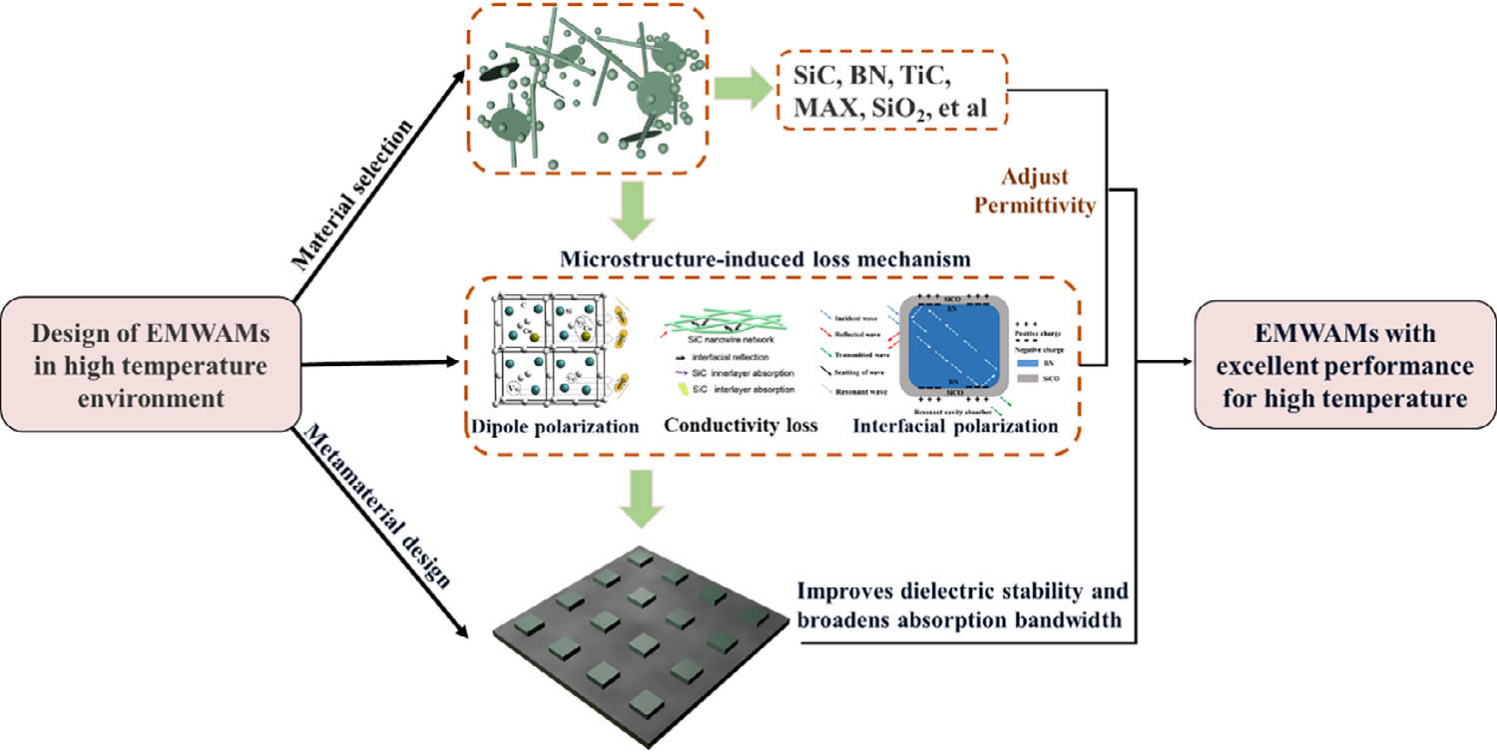
Design strategies for high‐temperature EMWAMs. Reproduced with permission.^[^ ^137^, ^141^, ^142^ ^]^ Copyright 2018, Elsevier. Copyright 2021, ACS. Copyright 2019, Elsevier.

According to the design requirements of electromagnetic wave absorbing materials in high‐temperature environments, it is necessary to construct a systematic design framework with a dielectric loss mechanism as the core. As shown in **Figure** **18**, the framework consists of three core design phases: screening of composite systems with high dielectric constant, excellent thermal stability, and oxidation resistance based on high‐temperature adaptability. Through the multicomponent collaborative design strategy, the dynamic balance of loss characteristics and structural stability is achieved in material composition and ratio, which lays a foundation for subsequent performance control. High‐temperature absorbing materials should focus on ceramic‐based materials, especially silicon carbide. Among them, silicon carbide fiber can provide strong interfacial polarization with its abundant stacking faults, and its dielectric constant is higher than that of other types of silicon carbide. By further constructing a variety of loss mechanisms, the electromagnetic wave absorption performance at high temperature can be effectively enhanced.

Nano/sub‐micrometer‐scale structural engineering for activating multiple loss mechanisms. Nano/sub‐micrometer‐scale structural engineering methods are used to activate multiple loss mechanisms such as interface polarization, conductivity loss, and Debye relaxation by regulating lattice defect density, constructing 3D conductive networks or designing heterogeneous interface structures. At this stage, special attention should be paid to the correlation and coupling of microstructural parameters and electromagnetic parameters, and the loss capacity should be exponentially enhanced through defect engineering and phase interface engineering.

Introduction of metamaterial designs concept for developing composite systems with periodic/quasiperiodic topology. Through the resonance coupling between geometric configuration and electromagnetic wave, the stability of dielectric parameters is maintained in high‐temperature environment, and the equivalent medium theory is used to expand the absorption frequency band. This stage focuses on breaking through the frequency band limitations of traditional materials and achieving collaborative optimization of a broad frequency band, strong absorption, and adaptability to high‐temperature environments.

Through the 3D collaborative innovation of materials, structures, and functions, this design framework systematically solves the key technical bottlenecks such as loss capacity attenuation, frequency band narrowing, and insufficient stability faced by electromagnetic wave absorbing materials in high‐temperature environments. Consequently, EMWAMs can achieve an electromagnetic wave energy absorption rate of more than 70% in the X‐band (8–12 GHz) in a wide temperature range from room temperature to 1000 °C.

## 5 Summary and Perspectives

A detailed analysis of the electromagnetic wave loss mechanisms of EMWAMs, particularly in high‐temperature environments, is introduced. The related testing methodologies are also discussed. Given the crucial role and urgent demand for EMWAMs capable of operating at temperatures above 800 °C for the critical positioning of hot‐end components in the aerospace field, this work summarizes several EMWAMs specifically designed for such high‐temperature conditions. However, this field still faces numerous challenges as follows:

1. With the pervasive application of electromagnetic waves in modern society, EMWAMs that can work stably at temperatures above 800 °C will be the focus of future research. Interfacial polarization loss represents a significant form of electromagnetic wave loss in high‐temperature environments; optimizing the interface structure of materials and modulating the interface polarization effect will be an important strategy to achieve the high‐performance goal of absorption intensity at high temperature.
2. The advancement of high‐temperature electromagnetic wave absorption performance testing technology and advanced characterization techniques is crucial for driving the progress of high‐temperature EMWAMs. Furthermore, the synergistic use of in situ testing technology and cross‐domain electromagnetic testing methodologies are anticipated to delve deeper into the high‐temperature absorption mechanisms and offer crucial insights for material optimization.
3. Given the suitable EMWAMs at high temperature, SiC‐based electromagnetic wave absorbing materials have emerged as the most promising candidates for applications in high‐temperature environments exceeding 800 °C based on the electromagnetic loss mechanisms. Future research endeavors will focus on enhancing their high‐temperature dielectric stability and expanding their absorption bandwidth. What is more, metamaterials also exhibit promising potential in this field.
4. To realize the long‐term, stable application and large‐scale promotion of high‐temperature absorbing materials, it is necessary to focus on breakthroughs in the following directions: on the one hand, developing dynamic testing devices to simulate real service environments and establish multifactor coupling electromagnetic performance evaluation standards including oxidation, corrosion, and thermal shock; on the other hand, optimization of low‐cost coating manufacturing processes to solve the cost bottleneck of large‐scale applications.

## Conflict of Interest

The authors declare no conflict of interest.
